# Analysis of Genomic Sequence Motifs for Deciphering Transcription Factor Binding and Transcriptional Regulation in Eukaryotic Cells

**DOI:** 10.3389/fgene.2016.00024

**Published:** 2016-02-23

**Authors:** Valentina Boeva

**Affiliations:** ^1^Centre de Recherche, Institut CurieParis, France; ^2^INSERM, U900Paris, France; ^3^Mines ParisTechFontainebleau, France; ^4^PSL Research UniversityParis, France; ^5^Department of Development, Reproduction and Cancer, Institut CochinParis, France; ^6^INSERM, U1016Paris, France; ^7^Centre National de la Recherche Scientifique UMR 8104Paris, France; ^8^Université Paris Descartes UMR-S1016Paris, France

**Keywords:** motif discovery, transcription factors, binding sites, position-specific scoring matrices, regulation of gene transcription, ChIP-seq, binding motif models

## Abstract

Eukaryotic genomes contain a variety of structured patterns: repetitive elements, binding sites of DNA and RNA associated proteins, splice sites, and so on. Often, these structured patterns can be formalized as motifs and described using a proper mathematical model such as position weight matrix and IUPAC consensus. Two key tasks are typically carried out for motifs in the context of the analysis of genomic sequences. These are: identification in a set of DNA regions of over-represented motifs from a particular motif database, and *de novo* discovery of over-represented motifs. Here we describe existing methodology to perform these two tasks for motifs characterizing transcription factor binding. When applied to the output of ChIP-seq and ChIP-exo experiments, or to promoter regions of co-modulated genes, motif analysis techniques allow for the prediction of transcription factor binding events and enable identification of transcriptional regulators and co-regulators. The usefulness of motif analysis is further exemplified in this review by how motif discovery improves peak calling in ChIP-seq and ChIP-exo experiments and, when coupled with information on gene expression, allows insights into physical mechanisms of transcriptional modulation.

## Introduction

A eukaryotic genome contains a variety of structured patterns. A far from exhaustive list of genomic patterns includes (i) tandem repeats and transposable elements, (ii) stretches of GC- or AT-rich sequences (e.g., CpG islands in mammalian genomes), (iii) binding sites of DNA associated proteins (e.g., transcription factor binding sites), (iv) splice sites, and (v) DNA and RNA binding sites of non-coding RNA molecules. Different patterns may overlap each other. Therefore, although this review is focused on motifs for transcription factor binding sites (TFBSs), we provide a short overview of other types of genomic patterns.

### Transcription factor binding sites (TFBSs)

**Figure 1:**
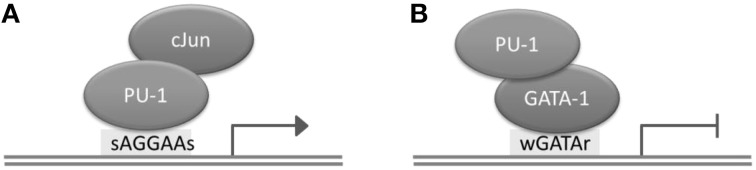
**Direct and indirect binding of TF PU.1 to DNA. (A)** Direct binding of PU.1 to DNA to the consensus motif sAGGAAs, which may lead to transcriptional activation. **(B)** Indirect binding of PU.1 to DNA, which may lead to transcriptional repression.

Transcription factors (TFs) are proteins with DNA binding activity that are involved in the regulation of transcription. Generally, TFs modulate gene expression by binding to gene promoter regions or to distal regions called enhancers. The distance between a TFBS and a transcription start site (TSS) of a gene regulated by the TF can be up to several megabases, and depends on the chromatin structure of the region (Dekker and Heard, 2015). Although TFs possess by definition DNA binding domains, they may occasionally bind DNA indirectly, by interacting with another TF. For instance, PU.1 and GATA-1 (TFs playing a critical role in the differentiation of hematopoietic lineages) interact through the ETS domain of PU.1 and the C-terminal finger region of TF GATA-1; as a result, PU.1 can bind to DNA both directly and indirectly, through the assistance of GATA-1 (Figure 1; Burda et al., 2010). A TF has binding preferences to a specific set of DNA sequences referred to as a “binding motif.” TFs have different binding affinities for sequences forming their binding motif set. Several mathematical models have been developed to represent a binding motif and take into account its properties. One of the most commonly used models is the positional weight matrix (PWM), also called the position-specific scoring matrix (PSSM), containing the log-odds or log-probability weights for computing the binding affinity score. Construction and use of the PWM model is discussed in detail in the next section. In some cases, the same TF is able to bind quite dissimilar motifs; the motif choice may predefine the action of this TF on gene expression (Guillon et al., 2009).

TFs often interact with each other or compete for DNA binding. Consequently, their binding sites may co-localize or overlap (Wang et al., 2012). Co-localization of TFBSs can be also due to the combined action of a set of TFs: First, TFs capable of binding inactive chromatin bind to DNA and create an open chromatin environment through the recruitment of histone acetyltransferases (pioneer TFs). Then, other TFs (lacking the above capability) become able to bind DNA and activate gene transcription by interacting with the RNA polymerase machinery (Farnham, 2009). Analysis of the distance and orientation preferences between the sites of co-binding TFs helps to predict possible protein-protein interactions, and enables insights into the mechanisms of transcriptional regulation by TFs when coupled with information on gene expression modulation.

### Repeats

Repeats constitute a large part of eukaryotic genomes. For instance, more than 45% of the human genome corresponds to repetitive sequences (Derrien et al., 2012). Among them, one distinguishes tandem repeats (DNA is repeated in head-to-tail fashion: microsatellites, minisatellites, and satellite sequences) and interspersed repeats (similar sequences are located throughout the genome). The latter correspond to transposable elements such as SINEs and LINEs, accounting for 12.5 and 20% of the human genome, respectively. Tandem repeats themselves account for 10–15% of the human genome. While short tandem repeats can serve as binding sites for specific transcription factors (TFs; Shi et al., 2000; Guillon et al., 2009), long satellite repeats can play a role in the 3D structure shaping of the genome. For instance, the α-satellite family of repeats (~171 bp tandem repeats) are bound by the fundamental component of the centromere CENP-C, and are essential for centromere function by ensuring proper chromosome segregation in mitosis and meiosis (Politi et al., 2002). The TandemSWAN software (http://favorov.bioinfolab.net/swan/tool.html) allows the annotation of exact and fuzzy tandem repeats in genomic sequences (Boeva et al., 2006). It is usual to mask such repeats in order to avoid artifact discovery, for example, during analysis of next-generation sequencing data.

### AT- or GC- rich sequences

AT- or GC- rich sequences are often located in gene promoters and play a role in transcription initiation. Approximately 24% of human genes contain an AT-rich sequence within the core promoter, with 10% containing a canonical TATA-box motif (TATAWAWR, W = A/T, R = A/G; Yang et al., 2007). The TATA-box recruits the TATA binding protein (TBP), which unwinds the DNA; also, due to weaker base-stacking interactions among A and T (than G and C), AT-rich sequences facilitate unwinding. The remaining 76% of human promoters are GC-rich and contain multiple binding sites of the transcriptional activator SP1 (Yang et al., 2007). As much as 56% of human genes, including most of the housekeeping genes, possess CpG islands, i.e., 300–3000 bp GC-rich sequences around gene TSS with a high density of CpG dinucleotides. The high methylation level of CpG sites in CpG islands has been shown to be associated with transcriptional repression. Polycomb group (PcG) repressor proteins recognize CpG islands that are unmethylated and unprotected by TFs (Klose et al., 2013). PcG proteins associate with DNA methyltransferases responsible for methylation of CpG islands (Viré et al., 2006). Also, some components of PcG proteins have histone methyltransferase activity and trimethylate histone H3 on lysine 27, which is a mark of transcriptionally silent chromatin.

### Splice site

During splicing, introns are removed from the pre-messenger RNA transcript and remaining exons are joined together to later form mature messenger RNA. Generally, in eukaryotes, the process of splicing is catalyzed by spliceosomes. These complex molecular machines recognize a donor site (almost invariably GU at the 5′ end of the intron), a branch site (adenine nucleotide followed by a pyrimidine-rich tract near the 3′ end of the intron), and an acceptor site (almost always AG at the 3′ end of the intron) on RNA transcripts. A DNA mutation in a splice site may have a wide range of functional consequences, among them exclusion of an exon from the mature mRNA, or inclusion of an intron or part of one. The latter often results in disruption of the reading frame or a premature stop codon, and thus gives rise to a defective or truncated protein.

### miRNA binding sites

While binding of regulatory proteins to promoter and enhancer DNA regions regulates expression of the targeted protein at the transcription level, binding of micro RNA molecules (miRNAs) to the 3′UTR region of a mRNA transcript can regulate the protein amount at the post-transcriptional level. The interaction of an miRNA as part of an active RNA-induced silencing complex (RISC) with a 3′UTR of the targeted mRNA transcript results in either inhibition of translation or increased degradation of this transcript. The miRNA complex recognizes the 6–8 nucleotides at the mRNA 3′UTR, which is complementary to the miRNA “seed” region (Bartel, 2009). In the human genome, there are more than 2000 unique miRNAs. One miRNA can target several genes, and the same 3′UTR can be targeted by multiple miRNAs. Sequence analysis of gene's 3′UTR, coupled with the analysis of evolutionary conservation of the 3′UTR region, allows the prediction of miRNA-target pairs (Yue et al., 2009). Mutations in an miRNA target site may disrupt miRNA repressive regulation, and thus result in protein overexpression (Chin et al., 2008). Alternatively, a mutation in the 3′UTR of a gene can create a new active miRNA binding site, negatively affecting gene expression (Ramsingh et al., 2010).

In this review, we present methods for *in silico* prediction of TFBSs, which can overlap any other type of genomic motif: repeats, CpG islands, splice sites, and so on. Some of the motif analysis methods discussed in this review in Section “*In silico* Detection of TFBSs” can be also applied to other types of motifs than TFBSs. In Section “Applications of Motif Analysis”, we also demonstrate how motif discovery can be used to improve peak calling from chromatin immunoprecipitation (ChIP) sequencing data and obtain insights about mechanisms of transcriptional regulation by specific TFs.

## *In silico* detection of transcription factor binding sites

We define TF binding motifs as sets of DNA sequences having high affinity for binding TFs. Each occurrence of a sequence from the binding motif in a genomic region is referred to as a motif instance. In the case of direct binding of a TF to DNA, a DNA region surrounding the binding site usually contains one or more instances of the corresponding binding motif.

There are several models for defining binding motifs. These can be used to scan a DNA sequence to predict TFBSs.

### Enumeration

All sequences with the potential to be bound by a TF can be enumerated. Information about these sequences can be obtained from SELEX experiments (Oliphant et al., 1989). To allow for discrimination between sequences with strong and weak binding affinities, one can use for example the SELEX affinity score assigned to each particular k-mer.

### Consensus

An alternative model for motif description is a consensus motif, constructed using the nomenclature of the International Union of Pure and Applied Chemistry (IUPAC):

**Table:** 

A = adenine	C = cytosine
G = guanine	T = thymine
Y = T | C (pyrimidine)	R = G | A (purine)
K = G | T (keto)	M = A | C (amino)
S = G | C (strong bonds)	W = A | T (weak bonds)
B = G | T | C (all but A)	V = G | C | A (all but T)
D = G | A | T (all but C)	H = A | C | T (all but G)
N = A | G | C | T (any)	

**Figure 2:**
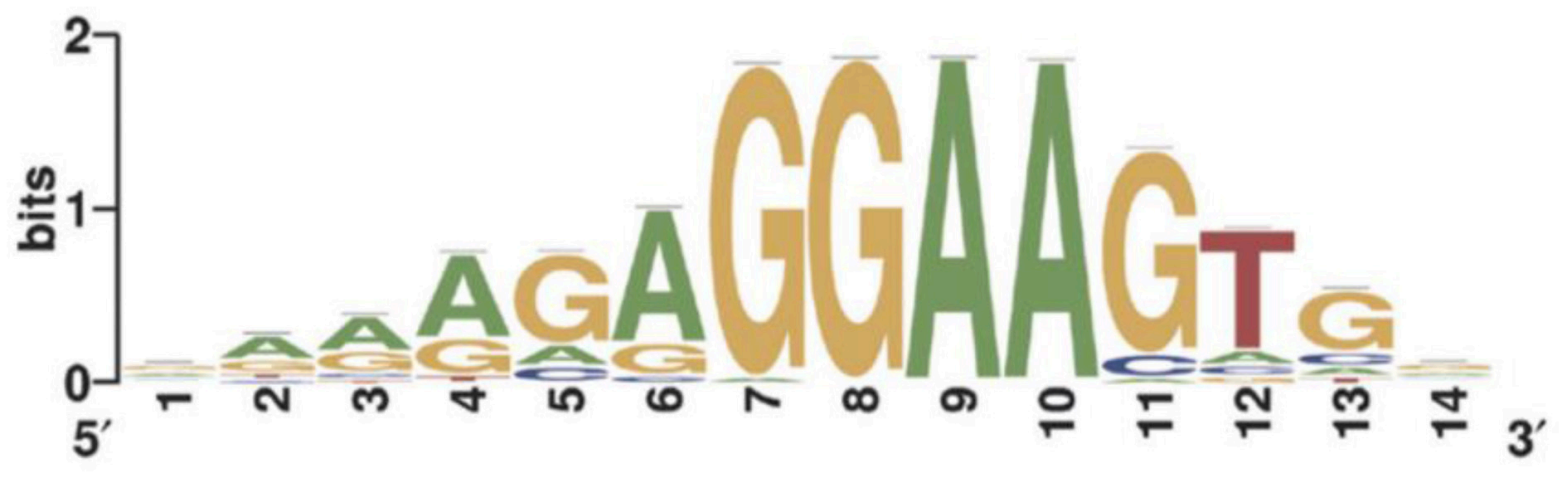
**Sequence logo of the PWM created by ChIPMunk (Kulakovskiy et al., 2010) using 17,781 binding site regions predicted for PU.1/Spi-1 using ChIP sequencing (ChIP-seq) data (Ridinger-Saison et al., 2012)**.

For instance, the IUPAC consensus for the binding motif of TF PU.1/Spi-1 can be written RRVRGGAASTS (the corresponding motif logo is depicted in Figure 2; Ridinger-Saison et al., 2012). The shortcoming of this way of modeling binding motifs is that many functional binding sequences may not be included in the motif when using a stringent consensus, and indeed, when consensus is poor, the motif can comprise motif instances of very low binding affinity, due to the uncaptured effect of nucleotide combinations on several low-affinity positions.

### Position weight matrix (PWM)

The PWM is the most frequently used mathematical model for binding motifs (Stormo, 2000). A PWM contains information about the position-dependent frequency or probability of each nucleotide in the motif. This information is usually represented as log-weights {*w*_α, *j*_} of probabilities (*w*_α, *j*_ = log(*p*_α, *j*_)) or, most frequently, odds ratios (*w*_α, *j*_ = *log*_2_(*p*_α, *j*_∕*b*_α_)) for computing a match score. Here *p*_α, *j*_ is the probability of nucleotide αα at position *j*, and *b*_α_ the background probability of nucleotide α. Small sample correction is usually included in *p*_α, *j*_ to avoid taking the logarithm of zero. A PWM match score for an arbitrary k-mer *A* = *a*_1_*a*_2_…*a*_*k*_ is computed as *S*_*A*_ = ∑_*j*_*w*_*a*_*j*__, *j*. Recent “deep learning” techniques (Alipanahi et al., 2015) use PWMs where weights are not required to be probabilities or log-odds ratios.

PWMs can be visualized using sequence logos (Schneider and Stephens, 1990; Figure 2). The total height of each bin is the information content in bits of the corresponding position: *H*_*j*_ = 2−∑_α_*p*_α_, *j*log_2_(*p*_α, *j*_). The height of each nucleotide in the logo is proportional to its probability *p*_α, *j*_ and, for each position, the four nucleotides are ordered by *p*_α, *j*_ with the most likely nucleotides depicted on top of the stack.

PWMs can be experimentally determined from SELEX experiments or computationally discovered from protein binding microarrays (PBMs; Berger and Bulyk, 2009), genomic-context PBM (gcPBM; Gordân et al., 2013), ChIP-seq, and ChIP-exo data.

Using the PWM motif representation, it is possible to distinguish strong binding sites (high PWM score) from weak binding sites (moderate PWM score). It may however, be a problem to discriminate weak binding sites from background (low or negative PWM score). Usually, a cutoff in the PWM score is used to decide whether a given sequence matches the motif. The choice of this cutoff is a complex statistical task that we discuss further here and in Section “Detection of TFBSs with Known PWMs”.

A PWM is constructed based on single nucleotide frequencies (four letter alphabet). However, from the methodological point of view, this model can be easily extended to the 16 letter alphabet of consecutive dinucleotides. This model has been used in the *de novo* motif discovery methods Dimont (Grau et al., 2013), diChIPMunk (Kulakovskiy I. et al., 2013), and BEEML-PBM (Zhao and Stormo, 2011; Zhao et al., 2012), the latter being designed to work with PBM data.

### Bayesian networks and other supervised classification methods

Although PWM is the most widely used mathematical representation of TF specificity, it still has drawbacks. For instance, it assumes the independence of positions within the motif: each position contributes separately to the PWM score, which reflects binding affinity. Modeling position dependencies with Bayesian networks provides an elegant solution to this problem (Barash et al., 2003; Ben-Gal et al., 2005; Grau et al., 2006). However, since there is no easy way to visualize motifs defined as a Bayesian network, this approach is rarely used by the research community.

This class of models was followed by another class of graphical model approaches based on Markov models (Wasson and Hartemink, 2009; Reid et al., 2010; Mathelier and Wasserman, 2013; Eggeling et al., 2014). The approach proposed by Mathelier and Wasserman (2013) has been included in the JASPAR database. Slim probabilistic graphical models, implemented by Keilwagen and Grau (2015), can be used via a Galaxy wrapper (http://galaxy.informatik.uni-halle.de); the authors also provide an intuitive model visualization.

In addition, motifs can be modeled and searched for using k-mer frequencies via support vector machine (SVM) approaches (Holloway et al., 2005; Jiang et al., 2007; Gorkin et al., 2012; Fletez-Brant et al., 2013). This class of approaches can be successfully applied to PBM data (Agius et al., 2010; Mordelet et al., 2013).

One of the important advantages of these graphical model and SVM-based approaches is that they can account for variable spacing between half-sites of two-box TFs (examples of such motifs are shown in **Figure 6A**). The DREAM5 challenge paper provides a comparative study of different methods for modeling transcription factor sequence specificity (Weirauch et al., 2013).

Given a motif described with one of the above-listed models, one can scan a set of genomic sequences or even a whole genome in order to detect possible TF binding sites. This can be achieved by applying efficient algorithms employing deterministic and non-deterministic finite automata accepting motif instances (Navarro and Raffinot, 2002; Antoniou et al., 2006; Boeva et al., 2007; Marschall and Rahmann, 2008; Marschall, 2011; Holub, 2012). The AhoPro (http://favorov.bioinfolab.net/ahokocc/seach_motifs.html Boeva et al., 2007) and PWMTools (http://ccg.vital-it.ch/pwmtools/pwmscan.php, Iseli et al., 2007) websites allow for fast online searches of instances of motifs with several of the models described above, in a set of sequences in FASTA format or in whole genomes. More tools allowing for a fast scan of sequences in FASTA format for motif instances are listed in the next section.

In the following, we choose the PWM model to represent binding motifs. Given that a cutoff is correctly selected, we assume that a TF binds DNA sequences with PWM scores higher than the cutoff. This assumption is a very rough approximation of reality. Using a high cutoff implies rejecting most of the weak binding sites, while using a lower cutoff can result in adding too much noise to predictions and muddle biological conclusions. In practice, the cutoff can be selected in a way to predict one motif instance per 1 or 10 Kb of the genome (Kulakovskiy I. V. et al., 2013). Cutoff choice can be also based on the hypothesis that the corresponding motif is over-represented in a given set of DNA sequences; this cutoff selection strategy is discussed in the next section.

*In silico* detection of TFBS may be separated into two tasks: detection of binding sites of TFs with known binding motifs (PWMs), and *de novo* motif discovery. Sections “Detection of TFBSs with Known PWMs” and “*De novo* Motif Discovery” focus on these two questions.

### Detection of TFBSs with known PWMs

Detection of TF binding motif instances for known motifs has its application in promoter analysis or the analysis of more distant regulatory regions (enhancers), where the goal is to find TFs possibly regulating corresponding genes. Scanning a set of sequences with PWMs of known motifs can also be used to detect co-factor binding in ChIP-seq-derived binding site regions of a TF of interest. Alternatively, one can use known-motif discovery to assess the effect of SNPs and mutations on TF binding. With the increase in the number of sequenced genomes, the second question has recently gained in importance, and novel tools permitting annotation of variants within TF motif instances have begun to be developed (Boyle et al., 2012; Ward and Kellis, 2016).

There exist several public and commercial databases storing PWMs for known TF binding motifs.

- HOCOMOCO: a comprehensive collection of human TFBS models (Kulakovskiy I. V. et al., 2013)
- JASPAR 2016: an extensively expanded and updated open-access database of TF binding profiles that can capture dinucleotide dependencies within TF binding sites (Mathelier et al., 2016)
- SwissRegulon: a database of genome-wide annotations of regulatory sites (Pachkov et al., 2007)
- TRANSFAC®: a commercial database on TFBSs, PWMs, and regulated genes in eukaryotes (Matys et al., 2006)
- footprintDB: a database summarizing motifs from HOCOMOCO, JASPAR, and other databases (Sebastian and Contreras-Moreira, 2014).

True binding sites usually score high with the corresponding PWM, while background sequences have low PWM scores. It is not sufficient to scan a DNA region to get a PWM score at each position. The main difficulty is to correctly set the cutoff on the PWM score to separate true binding sites from background. Evaluation of the statistical significance of motif instances can help solve this issue (Boeva et al., 2007).

When a PWM score cutoff *c* is given, it is possible to enumerate all possible sequences matching PWM with a score above the cutoff. Let us call this set *M*_*c*_ = {_*A*_*s*_1__, *A*_*s*_2__, …, *A*_*s*_*m*__}*s*_*i*_>*c*_, where each sequence *A*_*s*_*i*__ is a k-mer with PWM score *s*_*i*_ >*c*. The higher the cutoff *c*, the smaller the set of motif sequences *M*_*c*_. Given a set of regulatory regions (enhancers or promoters) *R*, we can define the number *N*_*R, c*_ showing how many *A*_*s*_*i*__ from *M*_*c*_ occurred in *R*. With a higher cutoff, fewer motif instances will be detected; corresponding binding sites are likely to have strong binding affinity. With a lower cutoff, more motif instances are detected; these may correspond to both strong and weak binding sites.

In regulatory regions, binding sites often tend to occur in clusters, and binding motifs are over-represented in the set *R* of regulatory sequences targeted by the transcription factor. This is not the case for random sequences. The procedure developed in Boeva et al. (2007) to specify the cutoff on the PWM score for a set *R* is based on this assumption.

**Figure 3:**
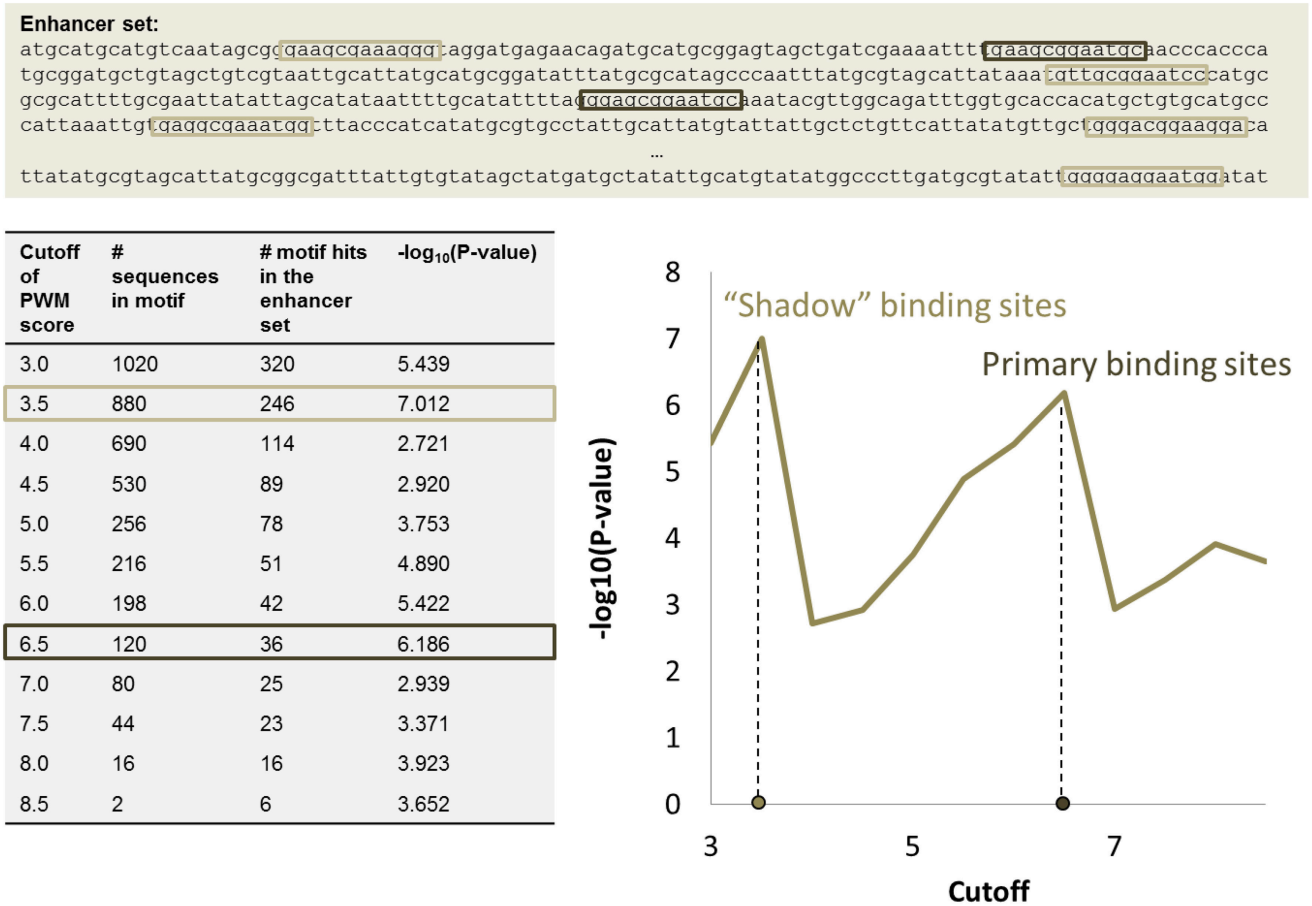
**PWM score cutoff selection for a set of enhancer regions**. Two local maxima in the *P*-value graph provide two *p*-value cutoffs that correspond to primary binding sites (high cutoff) and “shadow” binding sites (low cutoff). The table shows how many potential k-mer sequences match the PWM with a given cutoff (column 2), the number of motif instances in the set of enhancers (column 3), and the corresponding *p*-value (column 4).

**Figure 4:**
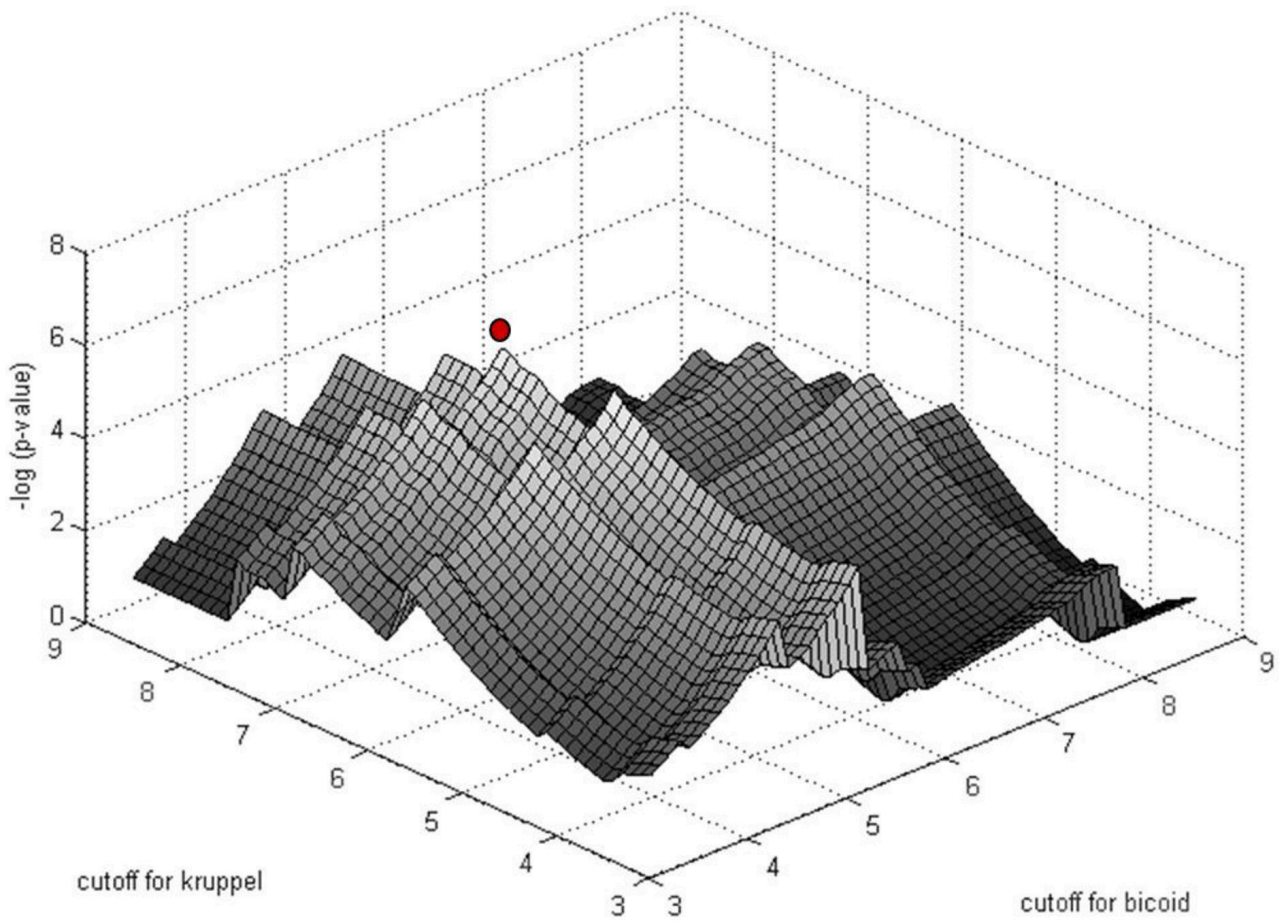
**Simultaneous PWM score cutoff selection for PWMs of two ***D***. ***melanogaster*** TFs: Bicoid and Krüppel**. The graph shows the distribution of log_10_(*p*-value) as a function of the cutoff for the two PWMs for the enhancer of the gene even-skipped stripe 2 (*eve2*). The red point corresponds to the most significant combination of PWM and cutoffs (from Boeva et al., 2007).

The significance of motif instance over-representation can be measured through the *p*-value, i.e., the probability to observe at least the same number *N*_*R, c*_ of motif instances with cutoff *c* in a random sequence with total length equal to the total length of sequences in *R* (Figure 3). Setting different cutoffs *c*, one gets different numbers of motif instances *N*_*R, c*_ in *R* and different *p*-values, P(*M*_*c*_, *N*_*R, c*_). The minimum of P(*M*_*c*_, *N*_*R, c*_) over *c* provides a cutoff corresponding to the most significant motif over-representation in R. This approach can be equally applied to several PWM corresponding to several TF binding motifs (Figure 4).

The exact *p*-value calculation for multiple motifs with overlapping (and self-overlapping) motifs is a difficult computational task. The compound Poisson distribution formula for the *p*-value generally provides a good approximation, but not in the case of several highly-overlapping motifs. An exact algorithm for *p-*value calculation for the general case of heterotypic clusters of motifs may be based on the Aho-Corasick automaton, and employ a prefix tree together with a transition function (Boeva et al., 2007; Marschall and Rahmann, 2008).

The approach for automatic cutoff choice for a set of PWMs was applied to the identification of binding sites of cooperatively and anti-cooperatively functioning regulatory proteins in *D. melanogaster* (Boeva et al., 2007). By employing this method, we discovered the phenomenon of “shadow” TFBS in enhancers of the *D. melanogaster* genome. Shadow binding sites are low affinity binding sites that alone are not capable of retaining the TF long enough to ensure activation/repression, but instead are used to maintain a high concentration of TF in the vicinity of the primary binding sites. This phenomenon has been recently confirmed by other studies (Kozlov et al., 2015).

We should mention that the choice of the background model is quite important in the calculation of probabilities of motif occurrences. A Markov chain employed as a background model allows us to capture dependencies between nucleotides. This can take into account low or high frequencies of CpG nucleotides in the set of enhancer or promoter sequences.

An automatic scan of a set of DNA sequences using motifs from the databases listed above, with tool-specific cutoffs, is available through the following websites and programs:

- AME or FIMO of the MEME suite (McLeay and Bailey, 2010) http://meme-suite.org/
- SeqPos of Galaxy Cistrome (Liu et al., 2011) http://cistrome.org/ap/
- PWMScan of PWMTools (Iseli et al., 2007) http://ccg.vital-it.ch/pwmtools/pwmscan.php
- oPOSSUM-3 (Kwon et al., 2012) http://opossum.cisreg.ca/oPOSSUM3/
- HOMER (Heinz et al., 2010) http://homer.salk.edu/homer/

### *De novo* motif discovery

When the PWM of a TF of interest is not known, it can be obtained using *de novo* motif discovery from a set of DNA sequences containing binding sites of this TF. The technique consists of defining the most over-represented motif in a given set of DNA sequences. The set of DNA sequences containing TFBSs of a particular protein can be obtained with SELEX, PBM or ChIP-x experiments (i.e., ChIP-seq, ChIP-exo, ORGANIC, ChIP-on-chip). ChIP-Seq (Johnson et al., 2007), ChIP-exo (Rhee and Pugh, 2011), and ORGANIC (Kasinathan et al., 2014) consist of immunoprecipitation of DNA–protein complexes and sequencing of short ends of the immunoprecipitated DNA. These techniques provide enhanced resolution of binding regions compared to ChIP-on-chip, which is based on microarrays, and have almost replaced the latter. The ChIP-exo technique provides an even better resolution of binding sites than ChIP-seq, at the expense of a more elaborate library preparation protocol, including an exonuclease step. In this section, we focus on *de novo* motif discovery in ChIP-seq datasets.

ChIP-seq yields a set of genomic regions (also called peaks) that are thought to contain TFBSs. The output of a ChIP-seq experiment can include tens of thousands of peaks, some longer than 1000 bp. Each peak position has a weight reflecting how often a given DNA fragment was cross-linked with the protein of interest during the ChIP stage (coverage profiles).

There exist a large number of methods for the *de novo* detection of over-represented motifs. The classical tool, MEME (Bailey et al., 2009), was developed for motif discovery in a small number of short DNA sequences, and scales poorly to large ChIP-seq datasets. Subsequently, several methods were newly created to analyze large sets of sequences resulting from ChIP-seq experiments: HMS (Hu et al., 2010), cERMIT (Georgiev et al., 2010), ChIPMunk (Kulakovskiy et al., 2010), diChIPMunk (Kulakovskiy I. et al., 2013), MEME-ChIP (Machanick and Bailey, 2011), POSMO (Ma et al., 2012), XXmotif (Hartmann et al., 2013), FMotif (Jia et al., 2014), Dimont (Grau et al., 2013), RSAT (Medina-Rivera et al., 2015), and DeepBind (Alipanahi et al., 2015). The latter method uses increasingly popular “deep learning” techniques; however, it has only been tested on sets of rather short input sequences (up to 101 bp).

There is a tradeoff between the user-friendliness of these tools, speed, and accuracy of predictions. For instance, the use of dinucleotide frequencies and application of read coverage profiles (.wig files) as priors for motif locations, improves the quality of resulting motifs. Both options are supported by diChIPMunk (Kulakovskiy I. et al., 2013). Dimont (Grau et al., 2013) can also use dinucleotide sequences for PWM construction and take into account peak height information, i.e., number of reads supporting each putative binding region. However, the user may find it encumbering extracting coverage information from the ChIP-seq data. Also, dinucleotide PWMs can come across as illegible in biological publications. It appears that intuitive and fast online methods based on classical PWMs are generally in higher demand by biologists than more sophisticated methods. Indeed, speed is one of the key issues in this type of analysis. In this context, k-mer enumeration methods like POSMO (Ma et al., 2012), cERMIT (Georgiev et al., 2010), and RSAT-peak-motifs (Medina-Rivera et al., 2015) show very competitive runtimes on large ChIP-seq datasets. However, probabilistic approaches (e.g., ChIPMunk, Dimont) may provide higher accuracy results (Grau et al., 2013). Overall, according to comparative studies, POSMO, Dimont, and ChIPMunk seem to be the most suitable methods for motif discovery among currently available ones (Ma et al., 2012; Grau et al., 2013). However, a more detailed study including more recent methods is required. More information about recently published methods is available in several reviews (Tran and Huang, 2014; Lihu and Holban, 2015). Most of the above-cited methods allow detection of *several* over-represented motifs. Below, we illustrate *de novo* multiple motif discovery with the ChIPMunk tool.

**Figure 5:**
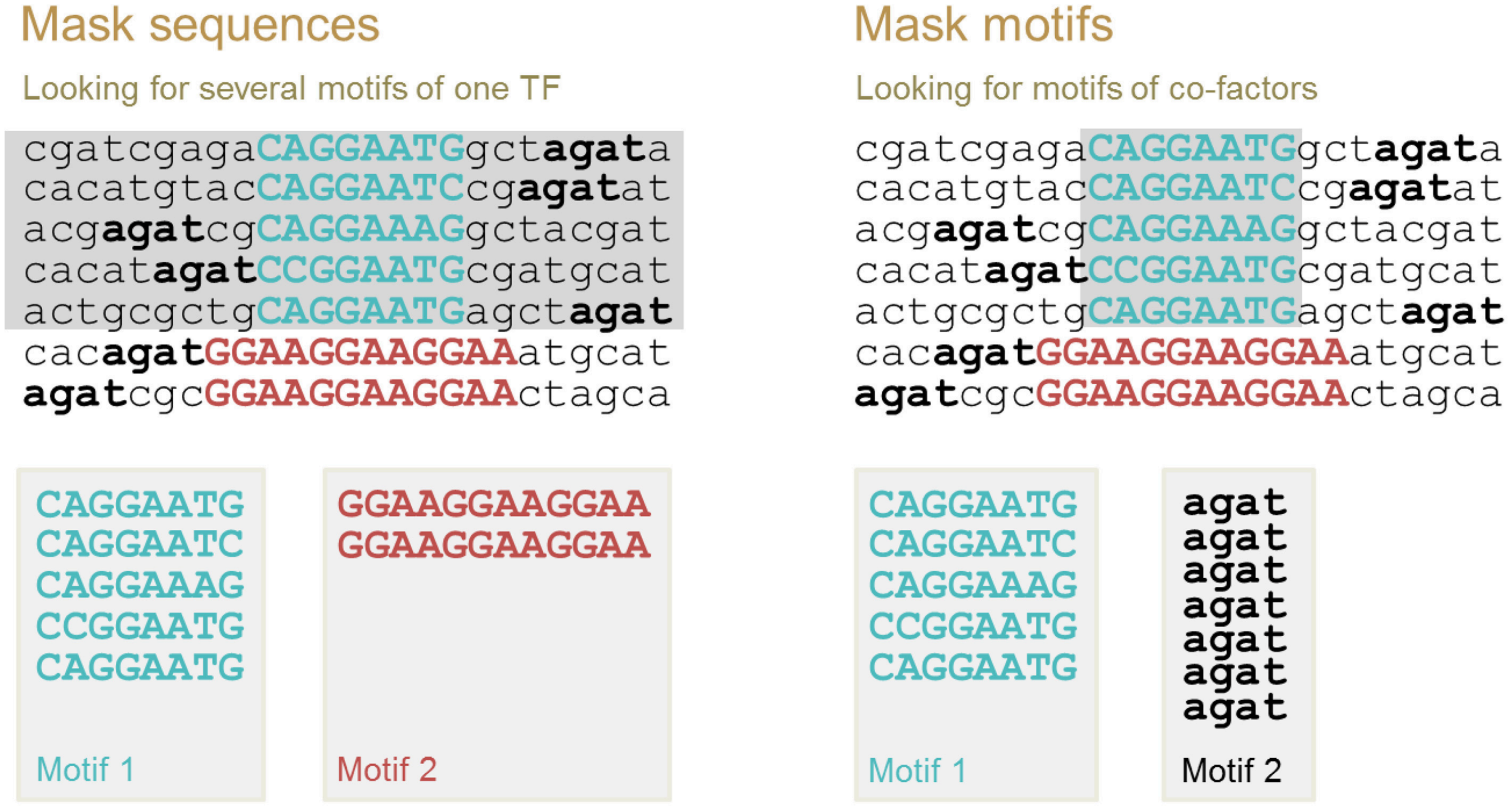
**Two modes of multiple motif detection: “Mask sequences” mode to discover binding motifs of the same TF, and “Mask motifs” mode to discover binding motifs of co-factors**. After the first motif is identified, either all sequences containing this motif instance are removed from further analysis (sequences in gray, “Mask sequences” mode), or motif instances are masked (motif instances in gray, “Mask motifs” mode). The second motif is defined as the motif with the highest KDIC in the remaining nucleotide sequences.

Multiple motif discovery allows us to identify (i) all possible binding motifs for the same TF and (ii) co-factor binding motifs. For these two cases, different motif discovery procedures should be applied. These two procedures are implemented in ChIPMunk as “Mask sequences” and “Mask motifs” modes. The first motif identified is always the motif with the highest Kullback discrete information content (KDIC). Then, the second motif is identified as the motif with the highest KDIC either in the sequences that do not contain the first motif (“Mask sequences” mode), or in the total set of sequences where the instances of the first motif have been masked (“Mask motifs” mode; Figure 5).

**Figure 6:**
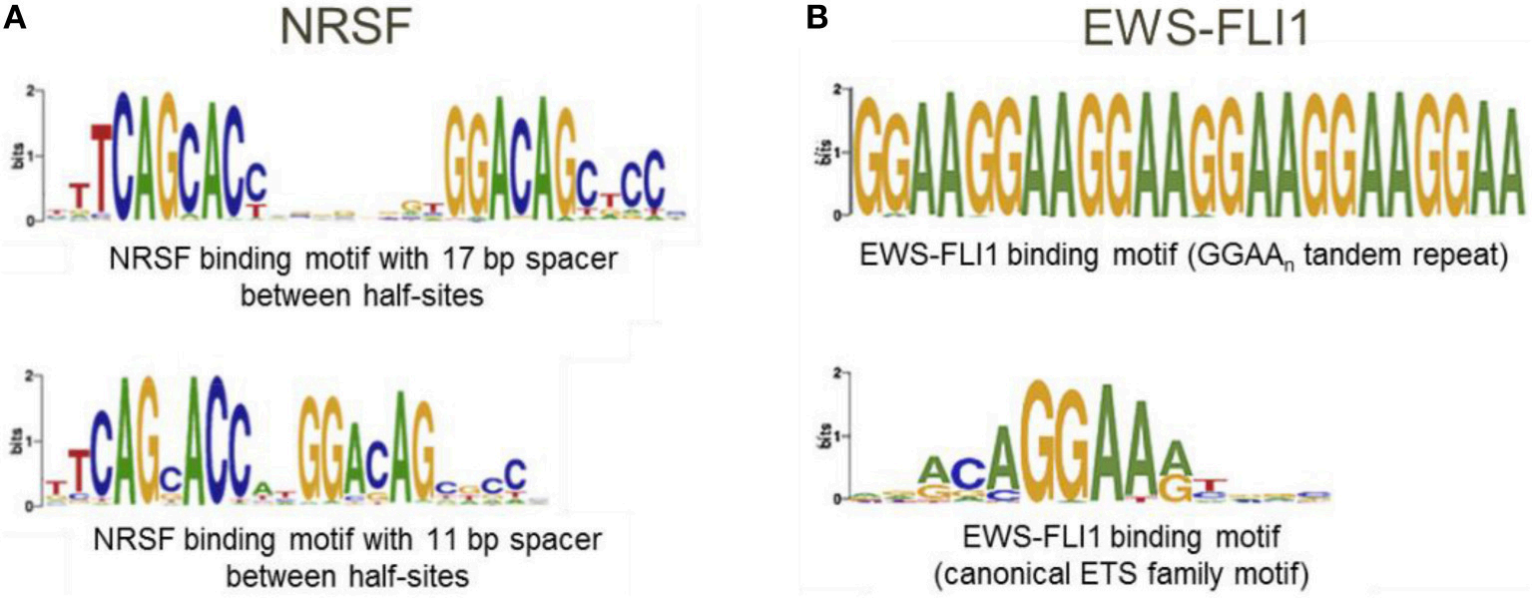
**A transcription factor can have several binding motifs. (A)** Logos for binding motifs of TF NRSF with 11 and 17 bp spacer between half-sites (Johnson et al., 2007); **(B)** Logos for binding motifs of chimeric TF EWS-FLI1 (Guillon et al., 2009; Boeva et al., 2010).

The underlying assumption when using the “Mask sequences” mode is that the same TF can, in some cases, bind to significantly different binding motifs; but almost every binding site region should contain at least one motif instance (Wang et al., 2012). We should mention that frequently a TF has only one binding motif; the higher the PWM score of the corresponding motif, the stronger the binding affinity (Kulakovskiy et al., 2010; Kulakovskiy I. V. et al., 2013). In this case, the “Mask sequences” mode is likely to output only one motif. This motif will be present in almost all sequences from the set. The situation where the same TF has different binding motifs, occur less frequently (Badis et al., 2009). For instance, this is the case for TFs EWS-FLI1 (Guillon et al., 2009) and NRSF (Johnson et al., 2007; Figure 6). Also, some proteins, such as PU.1, can bind to DNA both directly and indirectly (Figure 1). In these cases, the “Mask sequences” mode will provide, as a result, several motifs. This will be the motifs for the direct and indirect binding (e.g., motifs for PU.1 and GATA1 for the situation illustrated in Figure 2).

The underlying assumption for the use of the “Mask motifs” mode is that co-factors of the main TF bind close to the main TF in regions detected with chromatin immunoprecipitation using an antibody specific to the main TF of interest (Figure 5, right panel). Thus, binding motifs of co-factors can be detected as over-represented motifs after the motif instances of the main TF have been masked.

When a binding motif is identified *de novo*, it is possible to compare its PWM or IUPAC consensus with the known motif PWMs stored in the TF motif databases via:

- JASPAR (Mathelier et al., 2016)—http://jaspar.genereg.net/,
- Motif Comparison Tool of the MEME Suite (Gupta et al., 2007)—http://meme-suite.org/tools/tomtom
- MACRO-APE (Vorontsov et al., 2013)—http://autosome.ru/macroape/
- STAMP (Mahony and Benos, 2007)—http://www.benoslab.pitt.edu/stamp.

In this section, we have focused on the prediction of TFSB sites in a set of rather **short** regulatory regions provided by the user (regulatory regions obtained from ChIP-seq experiments). However, in some situations, one may be interested in analyzing much larger genomic regions (up to the whole genomes). In this case, one can narrow down the space of possible TFBS positions by considering known open chromatin regions in a given cell type, histone marks, and by using conservation profiles between species (Zhong et al., 2013). For instance, using a PWM-based score for the promoter, together with a profile of a single histone modification (H3K4me3), can produce highly accurate predictions of TF-promoter binding (McLeay et al., 2011).

## Applications of motif analysis

Motif discovery finds its applications in the analysis of promoters of co-expressed or co-regulated genes and in the analysis of regulatory regions frequently extracted from ChIP-x experiments. In this section, we explain a frequently applied procedure for promoter analysis. Then, we provide two examples on how motif analysis can be used in the exploration of ChIP-x data. We show how motif information can be applied to get a more accurate set of TFBSs from a ChIP-x experiment, and demonstrate how motif analysis can lead to insights into mechanisms of transcriptional regulation when it is integrated with information about changes in gene expression in a TF inhibition experiment.

### Promoter analysis: looking for over-represented TF motifs

Discovery of over-represented motifs in a set of genomic regions is often used to determine TFs likely to regulate genes co-modulated following some system perturbation, e.g., knockout or knockdown of a protein or cell differentiation. This type of study is called promoter analysis; it is based on the assumption that several promoters from the gene list are regulated by the same TF via binding of this TF to the promoter area of the corresponding genes. Thus, the goal of promoter analysis is to detect known (or less frequently *de novo*) motifs for which the number of motif instances is significantly higher in the set tested compared to background. As background, one should preferably use a set of promoters of non-modulated genes. Alternatively, one can define a set of random genomic regions or simply specify a background model (e.g., a Markov model of order 1 taking into account dinucleotide frequencies in promoters). Most of the methods apply the zero-or-one occurrences per sequence (ZOOPS) model (Bailey and Elkan, 1995), which enables detection of the strongest motif in a set of sequences; under this model, the strongest motif does not necessarily have instances in every input sequence. The presence of clusters of the same motif in one sequence is not taken into account by this model. The ZOOPS model is also applied by motif discovery tools designed to analyze ChIP-seq data (described above).

There are several major caveats to this approach. First, not every motif incidence corresponds to a true binding event. Thus, the definition of promoter length affects the results of the analysis. Larger promoter regions are likely to include a certain number of false predictions of binding sites, and at the same time are likely to capture more true binding sites. The use of large regions upstream of TSS in promoter analysis is especially unjustified when looking for short or highly degenerate motifs. The second caveat is that genes can be regulated by TF binding to distant regulatory elements: enhancers. These are often tissue specific, and thus not generally included in the set of sequences in which we look for motifs. The third caveat is the selection of the cutoff on the motif strength. Some methods allow the choice of the best cutoff as that providing the lowest *p*-value, while other methods use predefined cutoffs (Marstrand et al., 2008). Fourth, co-factors may be required for TF binding. In this case, one should probably search for combinations of motifs within a certain distance of one another.

Several tools have been developed specifically for promoter analysis. Some tools require gene lists while others expect sequences in FASTA format as input. The latter methods can be also applied to enhancer regions.

- Web-based promoter analysis tools:
  ◦ Amadeus (Linhart et al., 2008) http://acgt.cs.tau.ac.il/amadeus/—requires program download; can search for pairs of co-occurring motifs; accepts gene lists as input
  ◦ i-cisTarget (Herrmann et al., 2012; Imrichová et al., 2015) https://gbiomed.kuleuven.be/apps/lcb/i-cisTarget/—accepts.BED files or gene names; when gene names are provides, motif search is performed in 20 Kb window around gene TSSs overlapping with predefined candidate regularity regions
  ◦ Pscan (Zambelli et al., 2009) http://www.beaconlab.it/pscan—requires a gene list and provides a choice of 5 lengths for promoter intervals
  ◦ OTFBS (Zheng et al., 2003) http://genome.ucsf.edu/~jiashun/OTFBS/—online version accepts no more than 200 sequences in FASTA format
  ◦ Asap (Marstrand et al., 2008) http://servers.binf.ku.dk/asap/—accepts sequences in FASTA format; PWM threshold should be selected by the user
  ◦ oPOSSUM-3 (Kwon et al., 2012) http://opossum.cisreg.ca/oPOSSUM3/—accepts both sequences in FASTA format and gene lists
  ◦ Match and P-Match (Chekmenev et al., 2005) http://www.gene-regulation.com/pub/programs.html—TRANSFAC^®^ motif scanning algorithms
  ◦ SiTaR (Fazius et al., 2011) https://sbi.hki-jena.de/sitar/—needs a motif in enumeration format
- Offline promoter analysis tools:
  ◦ HOMER (Heinz et al., 2010)—command line tool to search for *de novo* motifs and compare them to known PWMs
  ◦ Clover (Frith et al., 2004).

The motifs in the output are sorted according to the method-specific *p*-values and enrichment scores. These *p*-values may be calculated through binomial or hyper-geometric statistical tests (Frith et al., 2004; Marstrand et al., 2008; Heinz et al., 2010; Kwon et al., 2012), ranking-and-recovery analysis of predefined tracks (Imrichová et al., 2015), or using the Z-transform of scores (Linhart et al., 2008; Zambelli et al., 2009). Correction for multiple tests is optionally performed by some methods (Marstrand et al., 2008).

As mentioned earlier, complementary information about sequence conservation, regions of open chromatin, and presence of specific histone marks, helps to increase TFBS prediction accuracy (Cuellar-Partida et al., 2012; Grant et al., 2015; Imrichová et al., 2015).

Promoter analysis usually predicts binding sites independently for several TFs. However, some recent approaches propose a different strategy, where the goal is to detect combinations of binding sites of several TFs forming cis-regulatory modules (CRMs). These approaches can be based on both *de novo* discovery of motifs, or using available motifs from databases. They can be applied to a set of promoter sequences, but also on predefined sets of enhancers, which can be obtained, for example, using profiles of histone marks. Some methods such as Allegro (Halperin et al., 2009) can take into account a range of changes in gene expression to better predict CRMs.

- Online tools:
  ◦ MatrixCatch (Deyneko et al., 2013) http://www.gene-regulation.com/cgi-bin/mcatch/MatrixCatch.pl—works with TFBS PWMs from the TRANSFAC^®^ database; accepts a set of sequences in FASTA format
  ◦ ModuleMiner (Loo et al., 2008) http://tomcatbackup.esat.kuleuven.be/moduleminer/—accepts Ensembl gene IDs to look for conserved CRMs upstream gene TSSs;
  ◦ PC-TraFF (Meckbach et al., 2015) http://pctraff.bioinf.med.uni-goettingen.de/—uses TRANSFAC^®^ PMWs on gene IDs or sequences in FASTA format
  ◦ DistanceScan (Shelest et al., 2010) https://www.omnifung.hki-jena.de/Rpad/Distance_Scan/index.htm—requires an output from FIMO or Match
  ◦ oPOSSUM-3 (Kwon et al., 2012) http://opossum.cisreg.ca/oPOSSUM3/—requires the name of the anchoring TF
  ◦ MCAST (Grant et al., 2015) http://meme-suite.org/tools/mcast—a tool from the extensive MEME suite; searches for clusters of provided motifs in sequences in FASTA format
  ◦ Cluster-Buster (Frith et al., 2003) http://zlab.bu.edu/cluster-buster/—searches for motif clusters; accepts PMWs in JASPAR or TRANSFAC^®^ formats
- Offline tools:
  ◦ ModuleDigger, CPModule, CORECLUST: stand-alone programs that require a set of known PWMs as input (Sun et al., 2009, 2012; Nikulova et al., 2012).

Validation of TFBSs can be carried out using a combination of chromatin immunoprecipitation with an antibody specific to the TF of interest, and real time PCR with primers specific to the predicted target region.

There are numerous illustrations of application of promoter analysis. For instance, analysis of promoters of protein coding genes and those of long non-coding RNA have shown that these two classes of genes tend to have different transcriptional regulators: motifs for 140 TFs were found to be over-represented in lncRNA gene promoters; this list of TFs includes nuclear hormone receptors and FOX family proteins (Alam et al., 2014). Dopamine-responsive genes have been shown to be regulated by the CREB protein (Frith et al., 2004). Analysis of melanocyte enhancers has predicted binding of key melanocyte TFs, including SOX10 and MITF (Gorkin et al., 2012). Motifs of 6 TFs (Hb, Foxa1, Cf2-ii, Lhx3, Mef2a, and slp1) have been found to be associated with insect bidirectional promoters (Behura and Severson, 2015). Similar analyses in the human genome have revealed 7 TFs (GABPA, MYC, E2F1, E2F4, NRF-1, CCAAT, and YY1) associated with promoter bidirectionality (Lin et al., 2007). Using promoter analysis, several ETS-domain TFs (GABPA, ELK1, and ELK4) have been discovered as likely regulators of breast cancer relevant sense-antisense gene pairs (Grinchuk et al., 2015).

### The use of motif information improves the accuracy of binding site detection in chiP-seq and chiP-exo data

ChIP-seq and ChIP-exo (ChIP-x) experiments have been widely used to define genomic positions of TF binding and discover TF binding motifs. The usual way to process ChIP-x data is to define TF binding regions first, then perform motif discovery to construct PWMs of TF binding motifs. In this section, we show that simultaneous instead of successive analysis of ChIP-x signal and motif instances improves the accuracy of TFBS prediction (Boeva et al., 2010; Guo et al., 2012; Starick et al., 2015). Below, we briefly describe the main elements of ChIP-x data analysis.

In the first step of ChIP-x data analysis, by extending each read to the length of the initial immunoprecipitated DNA fragment, it is possible to identify areas of fragment overlap and locate candidate regions of TF-DNA binding. These regions with high fragment density are called candidate peaks (Fejes et al., 2008). Not every peak contains a true binding site. Low peaks (with moderate read density) can appear by chance. Thus, to characterize the read enrichment and discriminate true binding from background noise, a statistical model needs to be applied. There are more than 20 different tools that perform this task for ChIP-x TF data (Wilbanks and Facciotti, 2010; Kim et al., 2011). The background model may be based on the uniform distribution of sequenced reads along the genome. Under such a background model, a Poisson test can be applied to evaluate the significance of read over-representation in a given region (Zhang et al., 2008). Often, in the ChIP-seq protocol, a negative control experiment is performed to assess the distribution of sequenced reads in the background. Recent studies have shown that an appropriate control data set is critical for analysis of any ChIP-seq experiment, because of biases in DNA breakage during sonication (Landt et al., 2012). The ChIP-exo datasets are usually generated with negative controls.

**Figure 7:**
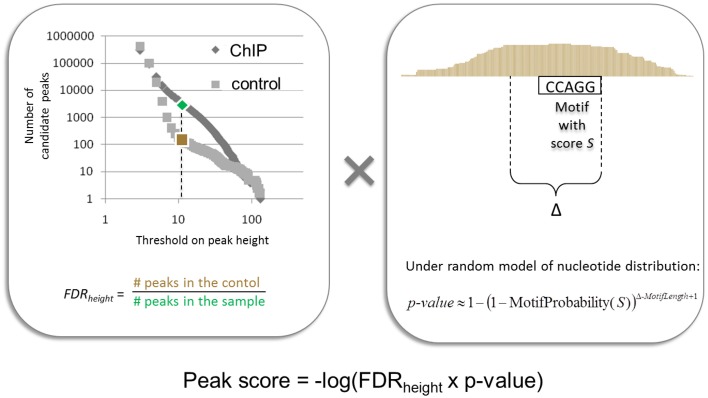
**Illustration of the procedure for peak score calculation used by the MICSA algorithm**.

In (Boeva et al., 2010), we presented a peak and motif calling algorithm, MICSA, based on the idea that functional binding sites of TFs should contain a consensus motif (or a set of consensus motifs). The MISCA workflow consists of four phases: (i) identification of all candidate peaks using read extension, (ii) identification of binding motif PWMs from a subset of peaks, (iii) detection of motif instances in all candidate peaks, and (iv) optimization of the peak calling output by calculating statistics taking into account information about both motif instance and depth of coverage. Importantly, MICSA identifies *several* binding motifs. The statistics calculated by MICSA allow us to retain strong binding sites (i.e., regions with high numbers of overlapping fragments) as well as weak binding sites with strong motif instances in the peak center (Figure 7). Weak binding sites without strong motif instances are removed from the final dataset. When applied to a ChIP-seq dataset for oncogenic TF EWS-FLI1, MICSA identified two consensus motifs (Figure 6B): a (GGAA)_≥6_ microsatellite, and a motif corresponding to the consensus RCAGGAARY, further referred to as the ETS motif. Surprisingly, the ETS motif did not coincide with the FLI1 binding motif (CCGGAARY), although EWS-FLI1 and FLI1 make up the same DNA-binding domain. Further analysis revealed the tendency of sites bearing GGAA-microsatellites to activate the expression of neighboring genes (sites found from 150-kb upstream to 50-kb downstream of gene TSSs), while sites with the ETS motif do not seem to have a definite activator function. In fact, ETS-sites negatively affected gene expression when located in the 50-kb region downstream of the TSSs. When ETS sites were located further away from gene TSSs (within 1 Mb upstream or downstream), both activator and inhibitory action of EWS-FLI1 was observed. More recent research from (Riggi et al., 2014) has shown that EWS-FLI1 creates *de novo* enhancers when it binds to GGAA-microsatellites, and may disrupt existing regulatory elements of ETS family TFs when it binds to single ETS-sites.

The idea of simultaneous analysis of the ChIP-x read density signal and motif instances has been further developed by Guo et al. (2012). Their GEM algorithm consists of five main steps: (i) detect candidate binding regions, (ii) discover and cluster sets of enriched k-mers, (iii) generate a positional prior for peak calling using k-mer classes, (iv) predict binding sites with a k-mer-based positional prior, and (v) re-discover enriched k-mer clusters in peaks from (iv). On the one hand, by considering motif information, the GEM method gives a better spatial resolution of binding sites than other peak calling methods, also enabling it to resolve closely-spaced binding events. On the other hand, on 214 ENCODE ChIP-Seq experiments for 63 TFs, binding motifs discovered by GEM were overall closer to the expected ones compared to motifs discovered by other methods. In fact, in 15 cases out of 215, GEM outperformed both MEME and ChIPmunk. Using the output of GEM on ENCODE ChIP-seq data in five different cell lines, Guo et al. (2012) studied pairwise binding relationships between different TFs. As a result, 390 pairs of TFs were shown to have significant binding distance constraints within a 100 bp distance, including known interaction pairs MYC-MAX, FOS-JUN, and CTCF-YY1.

The concept of combining ChIP-exo read density with motif information has been employed in the ExoProfiler computational pipeline (Starick et al., 2015). ExoProfiler searches for both *de novo* motifs and known motifs from the JASPAR database. It then extracts regions in ChIP-seq peaks centered on motifs, and analyzes strand specific read density. By applying ExoProfiler to glucocorticoid receptor (GR) ChIP-exo data, Starick et al. (2015) discovered indirect binding of GR to DNA via cofactors (FOX proteins) and discovered a novel GR binding sequence (“combi motif”), at which a GR forms a heterodimer with other TFs (ETS or TEAD families) to activate transcription.

### Getting insights into physical mechanisms of transcriptional modulation: co-directional clustered binding of the oncogenic TF Spi-1/PU.1 modulates gene expressionin erythroleukemia

Spi-1/PU.1 belongs to the same ETS TF family as FLI1 (the DNA-binding partner of EWS in the gene fusion causing Ewing sarcoma). Spi-1/PU.1 expression beyond physiological expression levels promotes oncogenesis in erythroid cells (Rimmelé et al., 2010). Here, we refer to our study of Spi-1/PU.1 ChIP-seq data, where motif analysis allowed us to get insights into mechanisms of how Spi-1/PU.1 physically modulates the expression of its target genes (Ridinger-Saison et al., 2012).

Analysis of the Spi-1/PU.1 ChIP-seq dataset resulted in a total of 17,781 binding site regions, which were assigned to genes using the Nebula peak-to-gene annotation module (Boeva et al., 2012). Of the 21 Spi-1/PU.1 binding sites tested, 20 were validated using real time PCR. As we detected instances of the binding motif in 88% of the Spi-1/PU.1-bound regions, we concluded that in erythroleukemia, Spi-1/PU.1 binds to DNA directly.

Interestingly, bound to a gene or even to a gene promoter, Spi-1/PU.1 rarely causes transcriptional modulation. Half of all mouse genes contained Spi-1/PU.1 binding sites, i.e., within a −30 kb region upstream of the TSS to +5 kb downstream of the transcription end, but only 8.1% (854 out of 10,560) of the Spi-1/PU.1-occupied genes were transcriptionally modulated. Therefore, we decided to study what additional factors influenced the gene modulation activity of Spi-1/PU.1.

**Figure 8:**
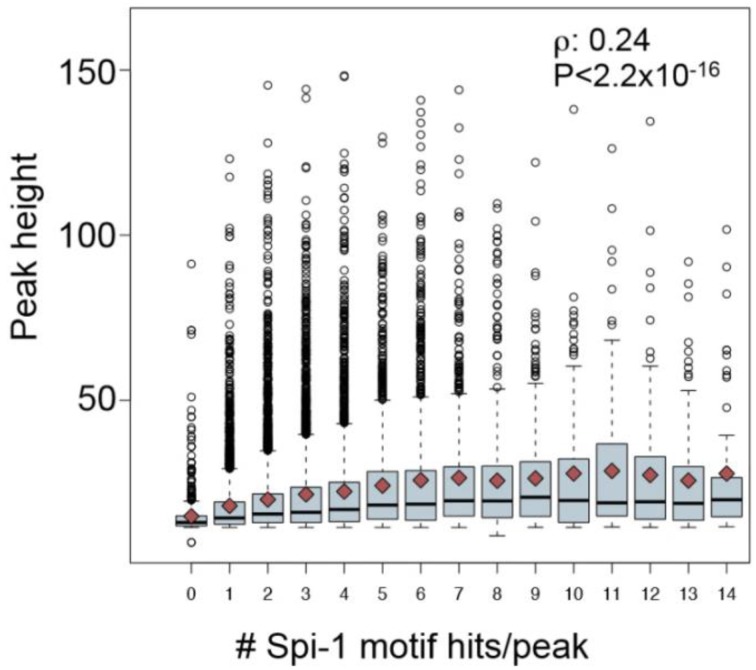
**The number of Spi-1/PU.1 motif instances correlates with the Spi-1/PU.1-binding intensity measured by the peak height**. The boxplot represents the distribution of the peak heights (y-axis) for each number of Spi-1/PU.1 motif instances/peak (x-axis). The dark red squares indicate the mean values, and the black line within each box indicates the median. The Spearman coefficient correlation (ρ) and the *p*-value of correlation test are reported (from Ridinger-Saison et al., 2012).

**Figure 9:**
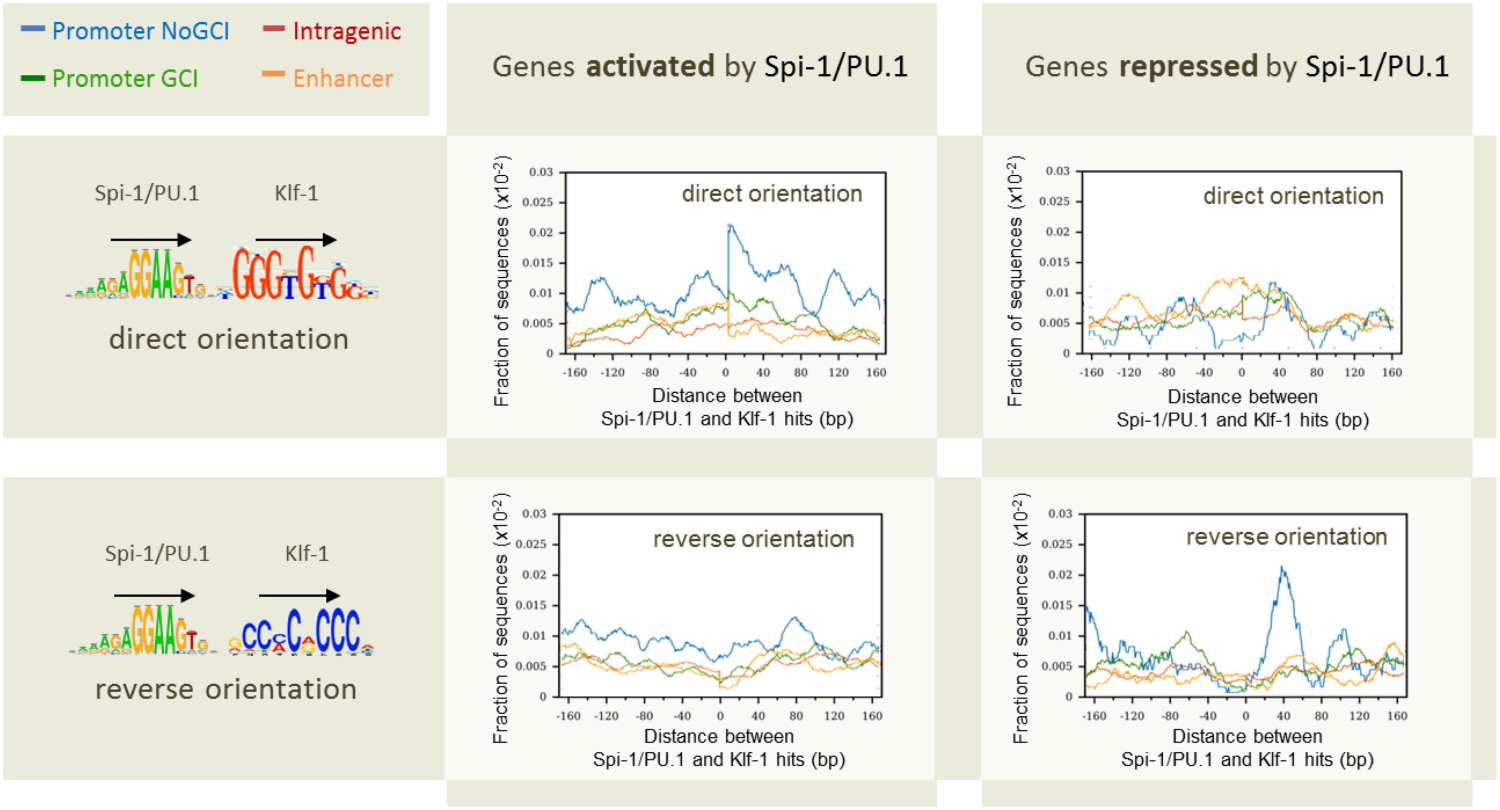
**Distribution of the distances between pairs of Spi-1/PU.1 and Klf-1 motif instances in direct or reverse orientation for genes activated and repressed by Spi-1/PU.1**. The x-axis shows the length of the spacers separating pairs of Spi-1 and Klf-1 motif instances. The y-axis shows the fraction of sequences with at least one pair of motif instances separated by the selected spacer. Promoters with CpG island (GCI): green; promoter devoted to CpG island (NoGCI): blue; enhancer regions: orange; intragenic regions: red. Data from Ridinger-Saison et al. (2012).

The first factor that correlated to the modulation status of genes was the distance between gene TSS and Spi-1/PU.1 binding sites: 60% of Spi-1/PU.1-activated genes contained Spi-1/PU.1 peaks in 5 kb area around TSSs, though only 40 and 22% of repressed and non-modulated genes, respectively, had peaks within this distance around TSSs. A second factor was the binding affinity, indicated by the peak height: peaks in the promoters of activated genes were significantly higher than in the promoters of repressed and non-modulated genes (*p*-value < 10^−5^). The binding affinity/peak height correlated with the number of motif instances per peak (Figure 8). In agreement with this observation, the number of Spi-1/PU.1 motif instances in Spi-1/PU.1 ChIP-seq peaks in promoters of activated genes was significantly higher than in promoters of repressed or non-modulated genes (*p*-values < 10^−6^). The third factor was the presence of a CpG island. Our analysis also indicated that Spi-1/PU.1 binding is favored at CG-rich sequences, but the absence of CpG islands increases the potential of Spi-1/PU.1 to activate gene expression. A fourth factor was the orientation of motif instances within a regulatory region. In cases when Spi-1/PU.1 induces gene modulation (activation or repression), Spi-1/PU.1 motif instances form co-oriented clusters (head-to-tail orientation). We observed these clusters of co-oriented motifs both in promoters of up-regulated genes, and enhancers of down-regulated genes. The fifth factor was the distance and orientation of Spi-1/PU.1 binding motifs, and motifs of other TFs. To get this information, we scanned ChIP-seq peak sequences with PWMs of known TFs using PATSER (Hertz and Stormo, 1999; Transfac and Jaspar motifs libraries). The most striking pattern was observed for pairs of Spi-1/PU.1 and KLF family motifs (Figure 9). For instance, in promoters of Spi-1/PU.1-up-regulated genes, we observed an enrichment of Spi-1/PU.1-KLF pairs where the direct KLF motif immediately follows the direct Spi-1/PU.1 motif. The patterns observed suggest cooperative interactions between Spi-1/PU.1 and KLF family TFs. The functional significance of these observations needs to be validated by biological experiments.

## Conclusion

Sequence analysis methods are extremely useful for decrypting the complex structure of patterns and motifs present in eukaryotic genomes. In particular, motif discovery methods applied to promoter/enhancer or ChIP-seq peak sequences enable detection of TFBSs in genomic DNA. In this review, we have presented *de novo* motif discovery techniques, and methods to find over-represented binding motifs of TFs with known motifs (PWMs). We have demonstrated that the application of these techniques improves accuracy of peak calling during ChIP-seq data analysis, and may provide novel biological insights into mechanisms of transcriptional regulation when sequence analysis is coupled with the analysis of gene expression changes. We expect that with time, motif discovery methods will become even more user-friendly, and will allow rapid processing of large datasets, while TRANSFAC®, JASPAR, and other databases will include an increasing number of TF motifs extracted from ChIP-seq experiments.

## Author contributions

The author confirms being the sole contributor of this work and approved it for publication.

## Acknowledgments

This work has been supported by The INSERM Atip-Avenir Program and The ARC Foundation.

### Conflict of interest statement

The author declares that the research was conducted in the absence of any commercial or financial relationships that could be construed as a potential conflict of interest.

## References

[B1] Agius P. Arvey A. Chang W. Noble W. S. Leslie C. (2010). High resolution models of transcription factor-DNA affinities improve *in vitro* and *in vivo* binding predictions. PLoS Comput. Biol. 6:e1000916. 10.1371/journal.pcbi.1000916 20838582 PMC2936517

[B2] Alam T. Medvedeva Y. A. Jia H. Brown J. B. Lipovich L. Bajic V. B. (2014). Promoter analysis reveals globally differential regulation of human long non-coding RNA and protein-coding genes. PLoS ONE 9:e109443. 10.1371/journal.pone.0109443 25275320 PMC4183604

[B3] Alipanahi B. Delong A. Weirauch M. T. Frey B. J. (2015). Predicting the sequence specificities of DNA- and RNA-binding proteins by deep learning. Nat. Biotechnol. 33, 831–838. 10.1038/nbt.3300 26213851

[B4] Antoniou P. Holub J. Iliopoulos C. S. Melichar B. Peterlongo P. (2006). Finding common motifs with gaps using finite automata, in Proceedings of the 11th International Conference on Implementation and Application of Automata CIAA'06 (Heidelberg: Springer-Verlag), 69–77.

[B5] Badis G. Berger M. F. Philippakis A. A. Talukder S. Gehrke A. R. Jaeger S. A.. (2009). Diversity and complexity in DNA recognition by transcription factors. Science 324, 1720–1723. 10.1126/science.1162327 19443739 PMC2905877

[B6] Bailey T. L. Boden M. Buske F. A. Frith M. Grant C. E. Clementi L.. (2009). MEME Suite: tools for motif discovery and searching. Nucleic Acids Res. 37, W202–W208. 10.1093/nar/gkp335 19458158 PMC2703892

[B7] Bailey T. L. Elkan C. (1995). The value of prior knowledge in discovering motifs with MEME. Proc. Int. Conf. Intell. Syst. Mol. Biol. 3, 21–29. 7584439

[B8] Barash Y. Elidan G. Friedman N. Kaplan T. (2003). Modeling dependencies in protein-DNA binding sites, in Proceedings of the Seventh Annual International Conference on Research in Computational Molecular Biology RECOMB'03 (New York, NY: ACM), 28–37.

[B9] Bartel D. P. (2009). MicroRNAs: target recognition and regulatory functions. Cell 136, 215–233. 10.1016/j.cell.2009.01.002 19167326 PMC3794896

[B10] Behura S. K. Severson D. W. (2015). Bidirectional promoters of insects: genome-wide comparison, evolutionary implication and influence on gene expression. J. Mol. Biol. 427, 521–536. 10.1016/j.jmb.2014.11.008 25463441 PMC4297529

[B11] Ben-Gal I. Shani A. Gohr A. Grau J. Arviv S. Shmilovici A.. (2005). Identification of transcription factor binding sites with variable-order Bayesian networks. Bioinformatics 21, 2657–2666. 10.1093/bioinformatics/bti410 15797905

[B12] Berger M. F. Bulyk M. L. (2009). Universal protein-binding microarrays for the comprehensive characterization of the DNA-binding specificities of transcription factors. Nat. Protoc. 4, 393–411. 10.1038/nprot.2008.195 19265799 PMC2908410

[B13] Boeva V. Clément J. Régnier M. Roytberg M. A. Makeev V. J. (2007). Exact p-value calculation for heterotypic clusters of regulatory motifs and its application in computational annotation of cis-regulatory modules. Algorithms Mol. Biol. 2:13. 10.1186/1748-7188-2-13 17927813 PMC2174486

[B14] Boeva V. Lermine A. Barette C. Guillouf C. Barillot E. (2012). Nebula—a web-server for advanced ChIP-seq data analysis. Bioinformatics 28, 2517–2519. 10.1093/bioinformatics/bts463 22829625

[B15] Boeva V. Regnier M. Papatsenko D. Makeev V. (2006). Short fuzzy tandem repeats in genomic sequences, identification, and possible role in regulation of gene expression. Bioinformatics 22, 676–684. 10.1093/bioinformatics/btk032 16403795

[B16] Boeva V. Surdez D. Guillon N. Tirode F. Fejes A. P. Delattre O.. (2010). *De novo* motif identification improves the accuracy of predicting transcription factor binding sites in ChIP-Seq data analysis. Nucleic Acids Res. 38, e126. 10.1093/nar/gkq217 20375099 PMC2887977

[B17] Boyle A. P. Hong E. L. Hariharan M. Cheng Y. Schaub M. A. Kasowski M.. (2012). Annotation of functional variation in personal genomes using RegulomeDB. Genome Res. 22, 1790–1797. 10.1101/gr.137323.112 22955989 PMC3431494

[B18] Burda P. Laslo P. Stopka T. (2010). The role of PU.1 and GATA-1 transcription factors during normal and leukemogenic hematopoiesis. Leukemia 24, 1249–1257. 10.1038/leu.2010.104 20520638

[B19] Chekmenev D. S. Haid C. Kel A. E. (2005). P-Match: transcription factor binding site search by combining patterns and weight matrices. Nucleic Acids Res. 33, W432–W437. 10.1093/nar/gki441 15980505 PMC1160202

[B20] Chin L. J. Ratner E. Leng S. Zhai R. Nallur S. Babar I.. (2008). A SNP in a let-7 microRNA complementary site in the KRAS 3′ untranslated region increases non–small cell lung cancer risk. Cancer Res. 68, 8535–8540. 10.1158/0008-5472.CAN-08-2129 18922928 PMC2672193

[B21] Cuellar-Partida G. Buske F. A. McLeay R. C. Whitington T. Noble W. S. Bailey T. L. (2012). Epigenetic priors for identifying active transcription factor binding sites. Bioinformatics 28, 56–62. 10.1093/bioinformatics/btr614 22072382 PMC3244768

[B22] Dekker J. Heard E. (2015). Structural and functional diversity of topologically associating domains. FEBS Lett. 589, 2877–2884. 10.1016/j.febslet.2015.08.044 26348399 PMC4598308

[B23] Derrien T. Estellé J. Marco Sola S. Knowles D. G. Raineri E. Guigó R.. (2012). Fast computation and applications of genome mappability. PLoS ONE 7:e30377. 10.1371/journal.pone.0030377 22276185 PMC3261895

[B24] Deyneko I. V. Kel A. E. Kel-Margoulis O. V. Deineko E. V. Wingender E. Weiss S. (2013). MatrixCatch - a novel tool for the recognition of composite regulatory elements in promoters. BMC Bioinformatics 14:241. 10.1186/1471-2105-14-241 23924163 PMC3754795

[B25] Eggeling R. Gohr A. Keilwagen J. Mohr M. Posch S. Smith A. D.. (2014). On the value of intra-motif dependencies of human insulator protein CTCF. PLoS ONE 9:e85629. 10.1371/journal.pone.0085629 24465627 PMC3899044

[B26] Farnham P. J. (2009). Insights from genomic profiling of transcription factors. Nat. Rev. Genet. 10, 605–616. 10.1038/nrg2636 19668247 PMC2846386

[B27] Fazius E. Shelest V. Shelest E. (2011). SiTaR: a novel tool for transcription factor binding site prediction. Bioinformatics 27, 2806–2811. 10.1093/bioinformatics/btr492 21893518

[B28] Fejes A. P. Robertson G. Bilenky M. Varhol R. Bainbridge M. Jones S. J. M. (2008). FindPeaks 3.1: a tool for identifying areas of enrichment from massively parallel short-read sequencing technology. Bioinformatics 24, 1729–1730. 10.1093/bioinformatics/btn305 18599518 PMC2638869

[B29] Fletez-Brant C. Lee D. McCallion A. S. Beer M. A. (2013). kmer-SVM: a web server for identifying predictive regulatory sequence features in genomic data sets. Nucleic Acids Res. 41, W544–W556. 10.1093/nar/gkt519 23771147 PMC3692045

[B30] Frith M. C. Fu Y. Yu L. Chen J.-F. Hansen U. Weng Z. (2004). Detection of functional DNA motifs via statistical over-representation. Nucleic Acids Res. 32, 1372–1381. 10.1093/nar/gkh299 14988425 PMC390287

[B31] Frith M. C. Li M. C. Weng Z. (2003). Cluster-buster: finding dense clusters of motifs in DNA sequences. Nucleic Acids Res. 31, 3666–3668. 10.1093/nar/gkg540 12824389 PMC168947

[B32] Georgiev S. Boyle A. P. Jayasurya K. Ding X. Mukherjee S. Ohler U. (2010). Evidence-ranked motif identification. Genome Biol. 11:R19. 10.1186/gb-2010-11-2-r19 20156354 PMC2872879

[B33] Gordân R. Shen N. Dror I. Zhou T. Horton J. Rohs R.. (2013). Genomic regions flanking E-box binding sites influence DNA binding specificity of bHLH transcription factors through DNA shape. Cell Rep. 3, 1093–1104. 10.1016/j.celrep.2013.03.014 23562153 PMC3640701

[B34] Gorkin D. U. Lee D. Reed X. Fletez-Brant C. Bessling S. L. Loftus S. K.. (2012). Integration of ChIP-seq and machine learning reveals enhancers and a predictive regulatory sequence vocabulary in melanocytes. Genome Res. 22, 2290–2301. 10.1101/gr.139360.112 23019145 PMC3483558

[B35] Grant C. E. Johnson J. Bailey T. L. Noble W. S. (2015). MCAST: scanning for cis-regulatory motif clusters. Bioinformatics btv750. [Epub ahead of print]. 10.1093/bioinformatics/btv750. 26704599 PMC4907379

[B36] Grau J. Ben-Gal I. Posch S. Grosse I. (2006). VOMBAT: prediction of transcription factor binding sites using variable order Bayesian trees. Nucleic Acids Res. 34, W529–W533. 10.1093/nar/gkl212 16845064 PMC1538886

[B37] Grau J. Posch S. Grosse I. Keilwagen J. (2013). A general approach for discriminative *de novo* motif discovery from high-throughput data. Nucleic Acids Res. 41, e197. 10.C/gkt831 24057214 PMC3834837

[B38] Grinchuk O. V. Motakis E. Yenamandra S. P. Ow G. S. Jenjaroenpun P. Tang Z.. (2015). Sense-antisense gene-pairs in breast cancer and associated pathological pathways. Oncotarget 6, 42197–42221. 10.18632/oncotarget.6255 26517092 PMC4747219

[B39] Guillon N. Tirode F. Boeva V. Zynovyev A. Barillot E. Delattre O. (2009). The oncogenic EWS-FLI1 protein binds *in vivo* ggaa microsatellite sequences with potential transcriptional activation function. PLoS ONE 4:e4932. 10.1371/journal.pone.0004932 19305498 PMC2654724

[B40] Guo Y. Mahony S. Gifford D. K. (2012). High resolution genome wide binding event finding and motif discovery reveals transcription factor spatial binding constraints. PLoS Comput. Biol. 8:e1002638. 10.1371/journal.pcbi.1002638 22912568 PMC3415389

[B41] Gupta S. Stamatoyannopoulos J. A. Bailey T. L. Noble W. S. (2007). Quantifying similarity between motifs. Genome Biol. 8:R24. 10.1186/gb-2007-8-2-r24 17324271 PMC1852410

[B42] Halperin Y. Linhart C. Ulitsky I. Shamir R. (2009). Allegro: analyzing expression and sequence in concert to discover regulatory programs. Nucleic Acids Res. 37, 1566–1579. 10.1093/nar/gkn1064 19151090 PMC2655690

[B43] Hartmann H. Guthöhrlein E. W. Siebert M. Luehr S. Söding J. (2013). P-value-based regulatory motif discovery using positional weight matrices. Genome Res. 23, 181–194. 10.1101/gr.139881.112 22990209 PMC3530678

[B44] Heinz S. Benner C. Spann N. Bertolino E. Lin Y. C. Laslo P.. (2010). Simple combinations of lineage-determining transcription factors prime cis-regulatory elements required for macrophage and B cell identities. Mol. Cell 38, 576–589. 10.1016/j.molcel.2010.05.004 20513432 PMC2898526

[B45] Herrmann C. Van de Sande B. Potier D. Aerts S. (2012). i-cisTarget: an integrative genomics method for the prediction of regulatory features and cis-regulatory modules. Nucleic Acids Res. 40, e114. 10.1093/nar/gks543 22718975 PMC3424583

[B46] Hertz G. Z. Stormo G. D. (1999). Identifying DNA and protein patterns with statistically significant alignments of multiple sequences. Bioinformatics 15, 563–577. 10.1093/bioinformatics/15.7.563 10487864

[B47] Holloway D. T. Kon M. DeLisi C. (2005). Integrating genomic data to predict transcription factor binding. Genome Inform. 16, 83–94. 16362910

[B48] Holub J. (2012). The finite automata approaches in stringology. Kybernetika 3, 386–401.

[B49] Hu M. Yu J. Taylor J. M. G. Chinnaiyan A. M. Qin Z. S. (2010). On the detection and refinement of transcription factor binding sites using ChIP-Seq data. Nucleic Acids Res. 38, 2154–2167. 10.1093/nar/gkp1180 20056654 PMC2853110

[B50] Imrichová H. Hulselmans G. Kalender Atak Z. Potier D. Aerts S. (2015). i-cisTarget 2015 update: generalized cis-regulatory enrichment analysis in human, mouse and fly. Nucleic Acids Res. 43, W57–W64. 10.1093/nar/gkv395 25925574 PMC4489282

[B51] Iseli C. Ambrosini G. Bucher P. Jongeneel C. V. (2007). Indexing Strategies for rapid searches of short words in genome sequences. PLoS ONE 2:e579. 10.1371/journal.pone.0000579 17593978 PMC1894650

[B52] Jia C. Carson M. B. Wang Y. Lin Y. Lu H. (2014). A new exhaustive method and strategy for finding motifs in ChIP-enriched regions. PLoS ONE 9:e86044. 10.1371/journal.pone.0086044 24475069 PMC3901781

[B53] Jiang B. Zhang M. Q. Zhang X. (2007). OSCAR: one-class SVM for accurate recognition of cis-elements. Bioinformatics 23, 2823–2828. 10.1093/bioinformatics/btm473 17921174

[B54] Johnson D. S. Mortazavi A. Myers R. M. Wold B. (2007). Genome-wide mapping of *in vivo* protein-DNA interactions. Science 316, 1497–1502. 10.1126/science.1141319 17540862

[B55] Kasinathan S. Orsi G. A. Zentner G. E. Ahmad K. Henikoff S. (2014). High-resolution mapping of transcription factor binding sites on native chromatin. Nat. Methods 11, 203–209. 10.1038/nmeth.2766 24336359 PMC3929178

[B56] Keilwagen J. Grau J. (2015). Varying levels of complexity in transcription factor binding motifs. Nucleic Acids Res. 43, e119–e119. 10.1093/nar/gkv577 26116565 PMC4605289

[B57] Kim H. Kim J. Selby H. Gao D. Tong T. Phang T. L.. (2011). A short survey of computational analysis methods in analysing ChIP-seq data. Hum. Genomics 5, 117–123. 10.1186/1479-7364-5-2-117 21296745 PMC3525234

[B58] Klose R. J. Cooper S. Farcas A. M. Blackledge N. P. Brockdorff N. (2013). Chromatin sampling—an emerging perspective on targeting polycomb repressor proteins. PLoS Genet. 9:e1003717. 10.1371/journal.pgen.1003717 23990804 PMC3749931

[B59] Kozlov K. Gursky V. V. Kulakovskiy I. V. Dymova A. Samsonova M. (2015). Analysis of functional importance of binding sites in the Drosophila gap gene network model. BMC Genomics 16(Suppl. 13):S7. 10.1186/1471-2164-16-S13-S7 26694511 PMC4686791

[B60] Kulakovskiy I. Levitsky V. Oshchepkov D. Bryzgalov L. Vorontsov I. Makeev V. (2013). From binding motifs in ChIP-Seq data to improved models of transcription factor binding sites. J. Bioinform. Comput. Biol. 11, 1340004. 10.1142/S0219720013400040 23427986

[B61] Kulakovskiy I. V. Boeva V. A. Favorov A. V. Makeev V. J. (2010). Deep and wide digging for binding motifs in ChIP-Seq data. Bioinformatics 26, 2622–2623. 10.1093/bioinformatics/btq488 20736340

[B62] Kulakovskiy I. V. Medvedeva Y. A. Schaefer U. Kasianov A. S. Vorontsov I. E. Bajic V. B.. (2013). HOCOMOCO: a comprehensive collection of human transcription factor binding sites models. Nucleic Acids Res. 41, D195–D202. 10.1093/nar/gks1089 23175603 PMC3531053

[B63] Kwon A. T. Arenillas D. J. Hunt R. W. Wasserman W. W. (2012). oPOSSUM-3: advanced analysis of regulatory motif over-representation across genes or ChIP-seq datasets. G3 2, 987–1002. 10.1534/g3.112.003202 22973536 PMC3429929

[B64] Landt S. G. Marinov G. K. Kundaje A. Kheradpour P. Pauli F. Batzoglou S.. (2012). ChIP-seq guidelines and practices of the ENCODE and modENCODE consortia. Genome Res. 22, 1813–1831. 10.1101/gr.136184.111 22955991 PMC3431496

[B65] Lihu A. Holban Ş. (2015). A review of ensemble methods for *de novo* motif discovery in ChIP-Seq data. Brief. Bioinformatics 16, 964–973. 10.1093/bib/bbv022 25888697

[B66] Lin J. M. Collins P. J. Trinklein N. D. Fu Y. Xi H. Myers R. M.. (2007). Transcription factor binding and modified histones in human bidirectional promoters. Genome Res. 17, 818–827. 10.1101/gr.5623407 17568000 PMC1891341

[B67] Linhart C. Halperin Y. Shamir R. (2008). Transcription factor and microRNA motif discovery: the Amadeus platform and a compendium of metazoan target sets. Genome Res. 18, 1180–1189. 10.1101/gr.076117.108 18411406 PMC2493407

[B68] Liu T. Ortiz J. A. Taing L. Meyer C. A. Lee B. Zhang Y.. (2011). Cistrome: an integrative platform for transcriptional regulation studies. Genome Biol. 12:R83. 10.1186/gb-2011-12-8-r83 21859476 PMC3245621

[B69] Loo P. V. Aerts S. Thienpont B. Moor B. D. Moreau Y. Marynen P. (2008). ModuleMiner - improved computational detection of cis-regulatory modules: are there different modes of gene regulation in embryonic development and adult tissues? Genome Biol. 9:R66. 10.1186/gb-2008-9-4-r66 18394174 PMC2643937

[B70] Ma X. Kulkarni A. Zhang Z. Xuan Z. Serfling R. Zhang M. Q. (2012). A highly efficient and effective motif discovery method for ChIP-seq/ChIP-chip data using positional information. Nucleic Acids Res. 40, e50–e50. 10.1093/nar/gkr1135 22228832 PMC3326300

[B71] Machanick P. Bailey T. L. (2011). MEME-ChIP: motif analysis of large DNA datasets. Bioinformatics 27, 1696–1697. 10.1093/bioinformatics/btr189 21486936 PMC3106185

[B72] Mahony S. Benos P. V. (2007). STAMP: a web tool for exploring DNA-binding motif similarities. Nucleic Acids Res. 35, W253–W258. 10.1093/nar/gkm272 17478497 PMC1933206

[B73] Marschall T. (2011). Construction of minimal deterministic finite automata from biological motifs. Theor. Comput. Sci. 412, 922–930. 10.1016/j.tcs.2010.12.003

[B74] Marschall T. Rahmann S. (2008). Probabilistic arithmetic automata and their application to pattern matching statistics, in Combinatorial Pattern Matching Lecture Notes in Computer Science, eds. Ferragina P. Landau G. M. (Heidelberg: Springer), 95–106. Available online at: http://link.springer.com.gate2.inist.fr/chapter/10.1007/978-3-540-69068-9_11 (Accessed December 21, 2015).

[B75] Marstrand T. T. Frellsen J. Moltke I. Thiim M. Valen E. Retelska D.. (2008). Asap: a framework for over-representation statistics for transcription factor binding sites. PLoS ONE 3:e1623. 10.1371/journal.pone.0001623 18286180 PMC2229843

[B76] Mathelier A. Fornes O. Arenillas D. J. Chen C.-Y. Denay G. Lee J.. (2016). JASPAR 2016: a major expansion and update of the open-access database of transcription factor binding profiles. Nucleic Acids Res. 44, D110–D115. 10.1093/nar/gkv1176 26531826 PMC4702842

[B77] Mathelier A. Wasserman W. W. (2013). The next generation of transcription factor binding site prediction. PLoS Comput. Biol. 9:e1003214. 10.1371/journal.pcbi.1003214 24039567 PMC3764009

[B78] Matys V. Kel-Margoulis O. V. Fricke E. Liebich I. Land S. Barre-Dirrie A.. (2006). TRANSFAC and its module TRANSCompel: transcriptional gene regulation in eukaryotes. Nucleic Acids Res. 34, D108–D110. 10.1093/nar/gkj143 16381825 PMC1347505

[B79] McLeay R. C. Bailey T. L. (2010). Motif Enrichment Analysis: a unified framework and an evaluation on ChIP data. BMC Bioinformatics 11:165. 10.1186/1471-2105-11-165 20356413 PMC2868005

[B80] McLeay R. C. Leat C. J. Bailey T. L. (2011). Tissue-specific prediction of directly regulated genes. Bioinformatics 27, 2354–2360. 10.1093/bioinformatics/btr399 21724591 PMC3157924

[B81] Meckbach C. Tacke R. Hua X. Waack S. Wingender E. Gültas M. (2015). PC-TraFF: identification of potentially collaborating transcription factors using pointwise mutual information. BMC Bioinformatics 16:400. 10.1186/s12859-015-0827-2 26627005 PMC4667426

[B82] Medina-Rivera A. Defrance M. Sand O. Herrmann C. Castro-Mondragon J. A. Delerce J.. (2015). RSAT 2015: regulatory sequence analysis tools. Nucleic Acids Res. 43, W50–W56. 10.1093/nar/gkv362 25904632 PMC4489296

[B83] Mordelet F. Horton J. Hartemink A. J. Engelhardt B. E. Gordân R. (2013). Stability selection for regression-based models of transcription factor–DNA binding specificity. Bioinformatics 29, i117–i125. 10.1093/bioinformatics/btt221 23812975 PMC3694650

[B84] Navarro G. Raffinot M. (2002). Flexible Pattern Matching in Strings: Practical On-line Search Algorithms for Texts and Biological Sequences. New York, NY: Cambridge University Press.

[B85] Nikulova A. A. Favorov A. V. Sutormin R. A. Makeev V. J. Mironov A. A. (2012). CORECLUST: identification of the conserved CRM grammar together with prediction of gene regulation. Nucleic Acids Res. 40, e93. 10.1093/nar/gks235 22422836 PMC3384346

[B86] Oliphant A. R. Brandl C. J. Struhl K. (1989). Defining the sequence specificity of DNA-binding proteins by selecting binding sites from random-sequence oligonucleotides: analysis of yeast GCN4 protein. Mol. Cell. Biol. 9, 2944–2949. 10.1128/MCB.9.7.2944 2674675 PMC362762

[B87] Pachkov M. Erb I. Molina N. van Nimwegen E. (2007). SwissRegulon: a database of genome-wide annotations of regulatory sites. Nucleic Acids Res. 35, D127–D131. 10.1093/nar/gkl857 17130146 PMC1716717

[B88] Politi V. Perini G. Trazzi S. Pliss A. Raska I. Earnshaw W. C.. (2002). CENP-C binds the alpha-satellite DNA *in vivo* at specific centromere domains. J. Cell. Sci. 115, 2317–2327. Available online at: http://jcs.biologists.org/content/115/11/2317.long 12006616 10.1242/jcs.115.11.2317

[B89] Ramsingh G. Koboldt D. C. Trissal M. Chiappinelli K. B. Wylie T. Koul S.. (2010). Complete characterization of the microRNAome in a patient with acute myeloid leukemia. Blood 116, 5316–5326. 10.1182/blood-2010-05-285395 20876853 PMC3012545

[B90] Reid J. E. Evans K. J. Dyer N. Wernisch L. Ott S. (2010). Variable structure motifs for transcription factor binding sites. BMC Genomics 11:30. 10.1186/1471-2164-11-30 20074339 PMC2824720

[B91] Rhee H. S. Pugh B. F. (2011). Comprehensive Genome-wide Protein-DNA Interactions Detected at Single-Nucleotide Resolution. Cell 147, 1408–1419. 10.1016/j.cell.2011.11.013 22153082 PMC3243364

[B92] Ridinger-Saison M. Boeva V. Rimmelé P. Kulakovskiy I. Gallais I. Levavasseur B.. (2012). Spi-1/PU.1 activates transcription through clustered DNA occupancy in erythroleukemia. Nucleic Acids Res. 40, 8927–8941. 10.1093/nar/gks659 22790984 PMC3467057

[B93] Riggi N. Knoechel B. Gillespie S. M. Rheinbay E. Boulay G. Suvà M. L.. (2014). EWS-FLI1 utilizes divergent chromatin remodeling mechanisms to directly activate or repress enhancer elements in ewing sarcoma. Cancer Cell 26, 668–681. 10.1016/j.ccell.2014.10.004 25453903 PMC4492343

[B94] Rimmelé P. Komatsu J. Hupé P. Roulin C. Barillot E. Dutreix M.. (2010). Spi-1/PU.1 oncogene accelerates DNA replication fork elongation and promotes genetic instability in the absence of DNA breakage. Cancer Res. 70, 6757–6766. 10.1158/0008-5472.CAN-09-4691 20660370

[B95] Schneider T. D. Stephens R. M. (1990). Sequence logos: a new way to display consensus sequences. Nucleic Acids Res. 18, 6097–6100. 10.1093/nar/18.20.6097 2172928 PMC332411

[B96] Sebastian A. Contreras-Moreira B. (2014). footprintDB: a database of transcription factors with annotated cis elements and binding interfaces. Bioinformatics 30, 258–265. 10.1093/bioinformatics/btt663 24234003

[B97] Shelest V. Albrecht D. Shelest E. (2010). DistanceScan: a tool for promoter modeling. Bioinformatics 26, 1460–1462. 10.1093/bioinformatics/btq132 20360058

[B98] Shi X. M. Blair H. C. Yang X. McDonald J. M. Cao X. (2000). Tandem repeat of C/EBP binding sites mediates PPARgamma2 gene transcription in glucocorticoid-induced adipocyte differentiation. J. Cell. Biochem. 76, 518–527. 10.1002/(SICI)1097-4644(20000301)76:3%3C518::AID-JCB18%3E3.0.CO;2-M 10649448

[B99] Starick S. R. Ibn-Salem J. Jurk M. Hernandez C. Love M. I. Chung H.-R.. (2015). ChIP-exo signal associated with DNA-binding motifs provides insight into the genomic binding of the glucocorticoid receptor and cooperating transcription factors. Genome Res. 25, 825–835. 10.1101/gr.185157.114 25720775 PMC4448679

[B100] Stormo G. D. (2000). DNA binding sites: representation and discovery. Bioinformatics 16, 16–23. 10.1093/bioinformatics/16.1.16 10812473

[B101] Sun H. De Bie T. Storms V. Fu Q. Dhollander T. Lemmens K.. (2009). ModuleDigger: an itemset mining framework for the detection of cis-regulatory modules. BMC Bioinformatics 10:S30. 10.1186/1471-2105-10-S1-S30 19208131 PMC2648767

[B102] Sun H. Guns T. Fierro A. C. Thorrez L. Nijssen S. Marchal K. (2012). Unveiling combinatorial regulation through the combination of ChIP information and *in silico* cis-regulatory module detection. Nucleic Acids Res. 40, e90–e90. 10.1093/nar/gks237 22422841 PMC3384348

[B103] Tran N. T. L. Huang C.-H. (2014). A survey of motif finding Web tools for detecting binding site motifs in ChIP-Seq data. Biol. Direct 9:4. 10.1186/1745-6150-9-4 24555784 PMC4022013

[B104] Viré E. Brenner C. Deplus R. Blanchon L. Fraga M. Didelot C.. (2006). The Polycomb group protein EZH2 directly controls DNA methylation. Nature 439, 871–874. 10.1038/nature04431 16357870

[B105] Vorontsov I. E. Kulakovskiy I. V. Makeev V. J. (2013). Jaccard index based similarity measure to compare transcription factor binding site models. Algorithms Mol. Biol. 8:23. 10.1186/1748-7188-8-23 24074225 PMC3851813

[B106] Wang J. Zhuang J. Iyer S. Lin X. Whitfield T. W. Greven M. C.. (2012). Sequence features and chromatin structure around the genomic regions bound by 119 human transcription factors. Genome Res. 22, 1798–1812. 10.1101/gr.139105.112 22955990 PMC3431495

[B107] Ward L. D. Kellis M. (2016). HaploReg v4: systematic mining of putative causal variants, cell types, regulators and target genes for human complex traits and disease. Nucleic Acids Res. 44, D877–D881. 10.1093/nar/gkv1340 26657631 PMC4702929

[B108] Wasson T. Hartemink A. J. (2009). An ensemble model of competitive multi-factor binding of the genome. Genome Res. 19, 2101–2112. 10.1101/gr.093450.109 19720867 PMC2775586

[B109] Weirauch M. T. Cote A. Norel R. Annala M. Zhao Y. Riley T. R.. (2013). Evaluation of methods for modeling transcription factor sequence specificity. Nat. Biotechnol. 31, 126–134. 10.1038/nbt.2486 23354101 PMC3687085

[B110] Wilbanks E. G. Facciotti M. T. (2010). Evaluation of algorithm performance in ChIP-seq peak detection. PLoS ONE 5:e11471. 10.1371/journal.pone.0011471 20628599 PMC2900203

[B111] Yang C. Bolotin E. Jiang T. Sladek F. M. Martinez E. (2007). Prevalence of the initiator over the TATA box in human and yeast genes and identification of DNA motifs enriched in human TATA-less core promoters. Gene 389, 52–65. 10.1016/j.gene.2006.09.029 17123746 PMC1955227

[B112] Yue D. Liu H. Huang Y. (2009). Survey of computational algorithms for microRNA target prediction. Curr. Genomics 10, 478–492. 10.2174/138920209789208219 20436875 PMC2808675

[B113] Zambelli F. Pesole G. Pavesi G. (2009). Pscan: finding over-represented transcription factor binding site motifs in sequences from co-regulated or co-expressed genes. Nucleic Acids Res. 37, W247–W252. 10.1093/nar/gkp464 19487240 PMC2703934

[B114] Zhang Y. Liu T. Meyer C. A. Eeckhoute J. Johnson D. S. Bernstein B. E.. (2008). Model-based analysis of ChIP-Seq (MACS). Genome Biol. 9:R137. 10.1186/gb-2008-9-9-r137 18798982 PMC2592715

[B115] Zhao Y. Ruan S. Pandey M. Stormo G. D. (2012). Improved models for transcription factor binding site identification using nonindependent interactions. Genetics 191, 781–790. 10.1534/genetics.112.138685 22505627 PMC3389974

[B116] Zhao Y. Stormo G. D. (2011). Quantitative analysis demonstrates most transcription factors require only simple models of specificity. Nat. Biotechnol. 29, 480–483. 10.1038/nbt.1893 21654662 PMC3111930

[B117] Zheng J. Wu J. Sun Z. (2003). An approach to identify over-represented cis-elements in related sequences. Nucleic Acids Res. 31, 1995–2005. 10.1093/nar/gkg287 12655017 PMC152803

[B118] Zhong S. He X. Bar-Joseph Z. (2013). Predicting tissue specific transcription factor binding sites. BMC Genomics 14:796. 10.1186/1471-2164-14-796 24238150 PMC3898213

